# Systemic ST6Gal-1 Is a Pro-survival Factor for Murine Transitional B Cells

**DOI:** 10.3389/fimmu.2018.02150

**Published:** 2018-09-20

**Authors:** Eric E. Irons, Joseph T. Y. Lau

**Affiliations:** Department of Molecular and Cellular Biology, Roswell Park Comprehensive Cancer Center, Buffalo, NY, United States

**Keywords:** B cell, humoral immunity, glycosylation, sialylation, ST6Gal-1, sialyltransferase

## Abstract

Humoral immunity depends on intrinsic B cell developmental programs guided by systemic signals that convey physiologic needs. Aberrant cues or their improper interpretation can lead to immune insufficiency or a failure of tolerance and autoimmunity. The means by which such systemic signals are conveyed remain poorly understood. Hence, further insight is essential to understanding and treating autoimmune diseases and to the development of improved vaccines. ST6Gal-1 is a sialyltransferase that constructs the α2,6-sialyl linkage on cell surface and extracellular glycans. The requirement for functional ST6Gal-1 in the development of humoral immunity is well documented. Canonically, ST6Gal-1 resides within the intracellular ER-Golgi secretory apparatus and participates in cell-autonomous glycosylation. However, a significant pool of extracellular ST6Gal-1 exists in circulation. Here, we segregate the contributions of B cell intrinsic and extrinsic ST6Gal-1 to B cell development. We observed that B cell-intrinsic ST6Gal-1 is required for marginal zone B cell development, while B cell non-autonomous ST6Gal-1 modulates B cell development and survival at the early transitional stages of the marrow and spleen. Exposure to extracellular ST6Gal-1 *ex vivo* enhanced the formation of IgM-high B cells from immature precursors, and increased CD23 and IgM expression. Extrinsic sialylation by extracellular ST6Gal-1 augmented BAFF-mediated activation of the non-canonical NF-kB, p38 MAPK, and PI3K/AKT pathways, and accelerated tyrosine phosphorylation after B cell receptor stimulation. *in vivo*, systemic ST6Gal-1 did not influence homing of B cells to the spleen but was critical for their long-term survival and systemic IgG levels. Circulatory ST6Gal-1 levels respond to inflammation, infection, and malignancy in mammals, including humans. In turn, we have shown previously that systemic ST6Gal-1 regulates inflammatory cell production by modifying bone marrow myeloid progenitors. Our data here point to an additional role of systemic ST6Gal-1 in guiding B cell development, which supports the concept that circulating ST6Gal-1 is a conveyor of systemic cues to guide the development of multiple branches of immune cells.

## Introduction

The humoral immune system is central to the successful management of infectious insults, through resolving established infections and generating long-term protection against future exposures. Although humoral insufficiency puts the host at risk, dysregulation of normal B cell function and development underlies autoimmune conditions such as lupus and rheumatoid arthritis (1–3). The balance between immune response and tolerance is predicated on the timely delivery and interpretation of systemic cues, conveyed directly by cell contact or indirectly through soluble secreted factors such as growth factors, cytokines, and chemokines. Within the bone marrow, immature B cells that successfully display the B cell receptor enter several transitional stages as they embark upon migration to the spleen (4). During these stages, auto-reactive clones are selectively depleted by exposure to self-antigens—a critical regulatory step in prevention of autoimmunity (5). However, as much as 90% of developing B cells may be deleted due to this process, greatly restricting the mature immune repertoire available for antigen recognition (6). Therefore, the mechanisms controlling transitional B cell survival and development are significant to both the treatment of autoimmunity and development of improved immunization strategies.

The sialyltransferase ST6Gal-1 catalyzes the addition of α2,6-sialic acid to Gal-β1,4-GlcNAc termini on cell surface and secreted glycans. ST6Gal-1 has been widely implicated in stemness (7, 8), integrin-mediated cellular adhesions (9), and radiation- and chemo- resistance (10, 11). In cancers, elevated ST6Gal-1 expression is often associated with poor prognosis (12, 13). Immunologically, mice with ST6Gal-1 insufficiency have exuberant inflammatory responses with excessive granulocyte infiltration in response to both T_H_1 and T_H_2 stimuli (14–16). Global loss of ST6Gal-1 also manifests as a humoral immunodeficiency, characterized by reduced responsiveness to B cell receptor cross-linking, diminished circulating IgM, and impaired antibody production to T-independent and T-dependent antigens (17). The B cell receptor (BCR) accessory siglec CD22 recognizes the α2,6-sialic acid produced by ST6Gal-1, and its disrupted engagement is thought to result in the humoral defects of ST6Gal-1 deficient animals (17, 18). Another B cell siglec, Siglec-10 (murine Siglec-G) also recognizes α2,6-sialic acids and may serve a similar function in the B1 lineage (19).

Canonically, glycosyltransferases such as ST6Gal-1 reside within the ER-Golgi complex and cell-autonomously glycosylate nascent glycoproteins destined for secretion or the cell surface. However, ST6Gal-1 is also released into extracellular spaces, and its abundance in systemic circulation is regulated dynamically by the liver in response to trauma and inflammation (20–23). In contrast to intracellular ST6Gal-1, secreted ST6Gal-1 can remodel glycans cell non-autonomously on distant cell surfaces in a process termed “extrinsic sialylation” (24, 25). Extrinsic sialylation by systemic ST6Gal-1 modifies bone marrow granulocyte/monocyte progenitors (GMP), inhibiting G-CSF induced signaling and further differentiation into committed granulocyte progenitors (GP) (26). In the periphery, inflammation and thrombotic events trigger extrinsic sialylation of immune cells (24, 25). Extrinsic STGal-1 also determines the sialylation status of the F_c_ region of circulating IgG, suppressing inflammation in FcγRII-expressing innate immune cells (27, 28). Surprisingly, it is a deficit in the systemic pool that drives the exaggerated granulocytic inflammation in ST6Gal-1 deficient mice, underscoring a non-redundant role for extrinsic ST6Gal-1 as a systemic signal modulating inflammation and immune responses.

Previous reports have described the transcriptional complexity in B cell-autonomous usage of ST6Gal-1 (29), with expression inducible by BCR activation and highest in mature and antibody-secreting subtypes (30). Before the recent understanding of the physiologic relevance of circulatory ST6Gal-1, it was presumed that the humoral defects accompanying ST6Gal-1 deficiency are due to an inability of B cells to natively express the enzyme. In this study, we report that ST6Gal-1 is required at two distinct developmental stages in B lymphopoiesis. ST6Gal-1 deficiency results in impaired marginal zone maturation, a phenotype noted previously (31), and our data show that this is due to impaired B cell-intrinsic ST6Gal-1 expression. A second impairment, in early transitional B cell development, is attributed to a requirement for B cell non-autonomous ST6Gal-1. Extrinsic sialylation by ST6Gal-1 enhances transitional B cell development, CD23 expression, BAFF and BCR-mediated pro-survival signaling, and survival after negative selection. Adoptive transfer studies in B cell deficient animals point to a role for systemic ST6Gal-1 in long-term survival of B cells. Taken together, the data point to not only the importance of B cell-intrinsic ST6Gal-1 in B cell development, but also the potential for B cell-extrinsic ST6Gal-1 to act as a systemic factor in guiding B lymphopoiesis to shape humoral immunity.

## Materials and methods

### Animal models

The *St6gal1*-KO strain used has been back-crossed 15 generations into C57BL/6J background and maintained at Roswell Park's Laboratory Animal Shared Resource (LASR) facility. The reference wild-type strain used was C57BL/6J from JAX. μMT mice (JAX) and μMT/ST6KO mice, generated by crossing single knockout strains, were used in adoptive transfer experiments. When specifically stated, the CD45.1 expressing strain, B6.SJL-Ptprc^a^ Pepc^b^/BoyJ, was used in order to distinguish donor cells from C57BL/6J, which expresses the CD45.2 allele of the Ptprc locus. For transplantations, mice received 6 Gy whole body gamma-radiation and were rescued with 4.0 × 10^6^ whole bone marrow cells. Mice were euthanized after 6 weeks for analysis. For B cell migration assays, splenocytes from 6 day-old wild-type mice were stained in 5 uM CFSE before intravenous transfer to recipients. For B cell survival assays, CD3-/B220+ splenocytes were intravenously transferred into recipients. Unless otherwise indicated, mice between 7 and 10 weeks of age were used, and both sexes were equally represented. Roswell Park Institute of Animal Care and Use Committee (IACUC) approved maintenance of animals and all procedures used under protocol 1071M.

### Antibodies

For flow cytometry, anti-B220-PE/Cy7 (RA3-6B2), anti-CD19-BV510 (GD5), anti-IgD-PE (11-26c.2a), anti-CD23-APC/Cy7 (B3B4), anti-IgM-APC (RMM-1), anti-CD21-PerCP/Cy5.5 (7E9), and anti-CD24-PE (30-F1) were purchased from Biolegend. For magnetic cell separation, biotinylated anti-IgM (RMM-1), anti-B220 (RA3-6B2), anti-Gr1 (RB6-8C5), anti-CD23 (B3B4), and anti-CD3e antibody (145-2C11) were purchased from BD Pharmingen. For microscopy, anti-α-smooth muscle actin (ab5694, Abcam), anti-IgM-Cy3 (EMD Millipore), anti-IgD-FITC (11-26c, Invitrogen), anti-MARCO-FITC (Bio-rad), and anti-B220 (RA3-6B2, eBioscience) were used. For western blot, anti-NFkB2, anti-p-p38 (T180/Y182), anti-pAkt (S473) from Cell Signaling Technology, anti-β-actin (Invitrogen), anti-BAFFR and anti-ST6Gal-1 (R&D Biosystems), and anti-pTyr (EMD Millipore) were used.

### Identification and analysis of cell populations

The parameters for flow cytometry visualization of B lineage populations were as follows. Bone marrow immature (IM: B220^low^/IgM^low^), IgM-hi (B220^+^/IgM^hi^), and bone marrow mature (BMM: B220^hi^/IgM^low−mid^); splenic IgD-/CD21-(B220^+^/CD19^+^/IgD^−^/IgM^+^/CD21^−^), IgD+/CD21+ (B220^+^/CD19^+^/IgD^+^/IgM^+^/CD21^+^), marginal zone (MZ: B220^+^/CD19^+^/IgD^−^/IgM^+^/CD21^+^), follicular (FO: B220^+^/CD19^+^/IgD^+^/IgM^low/mid^/CD21^mid^) B cells, and splenic plasma cells (SLPC: B220^−^/CD138^+^). Gating schemes are shown in Supplementary Figure 1, and gating controls in Supplementary Figure 9. Flow cytometry data was acquired with BD LSR II flow cytometer and analyzed with FlowJo software. FACS cell purification for RT-PCR and protein analysis was performed with BD FACS Aria II, yielding populations of >94% purity.

For RT-qPCR, sorted live cells were resuspended in TRI Reagent (MRC Inc.,) and RNA extracted according to manufacturer's instructions. 1.5 μg of RNA was converted to cDNA (iSCRIPT kit, Bio-rad), then amplified by qPCR (SYBR Green, Bio-rad) with intron-spanning primers. Primer sequences are as follows: B2M: F- 5′-CTGACCGGCCTGTATGCTAT-3′; R- 5′-TTCCCGTTCTTCAGCATTTGGAT-3′, ST6GAL1: F- 5′-CTTGGCCTCCAGACCTAGTAAAGT-3′; R- 5′-TCCCTTTCTTCCACACGCAGATGA-3′. Expression for St6gal1 was normalized to B2-microglobulin.

### Microscopy

Frozen spleens were sectioned onto glass slides. Slides were acetone fixed, rehydrated in PBS, then blocked in 5% BSA solution, followed by staining with antibodies per manufacturer's guidelines. Fluorescence was visualized immediately using a Nikon Eclipse E600 microscope with EXFO X-cite 120 light source. Spot RT3 camera and Spot Software were used to capture images.

### *Ex vivo* B cell culture and stimulation

Bone marrow from wild-type mice was depleted for IgM and Gr-1, then enriched for B220 by MACS columns (Miltenyi Biotechnology) for immature B cells (96% purity). Where indicated, B220+ IgM-low cells were cultured in RPMI with 10% non-mitogenic FBS and penicillin/streptomycin for 40 h. For B cell receptor (BCR) stimulation, CD23+ (rather than IgM+) cells were negatively selected to obtain immature and transitional B cells (~80% purity). For cell activation experiments, B cells were extrinsically sialylated with 40 μg/ml ST6Gal-1 and 0.05 mM CMP-sialic acid (Sigma C-8271) in serum-free RPMI for 2 h, then stimulated with 200 ng/ml murine BAFF (R&D Biosystems) or 10 μg/ml function-grade anti-IgM F(ab')2 (Invitrogen 16-5092-85). To model negative selection, cells were cultured at 1 × 10^5^ cells/ml as indicated in presence of 10 μg/ml ST6Gal-1, 0.05 mM CMP-sialic acid, 20 ng/ml BAFF, 10 μg/ml anti-IgM antibody, 1000 U/ml IL-4, and function-grade anti-CD40 antibody (eBioscience HM40-3) for 18–20 h. Live cells were quantified by DAPI flow cytometry. Recombinant rat secretory ST6Gal-1 was a generous gift from Dr. Kelley Moremen of the University of Georgia.

### Immunoblotting and immunoprecipitation

For western blots, indicated cells were lysed in NP-40 lysis buffer with protease and phosphatase inhibitors and immediately snap-frozen. Lysates were separated on 10% SDS-PAGE gels, transferred to PVDF membranes, and probed with primary antibodies overnight and secondary antibodies for 1 h. Membranes were developed using Pierce ECL WB Substrate (Thermo Scientific) and imaged using ChemiDoc Touch (Bio-rad). Where indicated, band intensity was quantified with ImageLab software. For immunoprecipitation, B cell membrane proteins were isolated using MEM-PER Plus kit (Thermo Scientific), then incubated with blocked SNA-agarose beads (Vector Laboratories) overnight. Beads were extensively washed and immunoprecipitate eluted by boiling in denaturing and reducing conditions, before western blot analysis. Uncropped Western blot images are included in Supplementary Figure 8.

### Serum immunoglobulin analysis

Detection of serum immunoglobulin G was achieved by ELISA (Bethyl Laboratories) according to manufacturer's protocols. Autoantigen-specific IgG was detected by direct ELISA against salmon sperm DNA, calf thymus histone, recombinant TPO (Cloud-Clone Corp.), or recombinant MPO (R&D Biosystems). Serum from the Ets-1 KO autoimmune mouse model was used as positive control (32). Calf thymus histone and Ets-1 KO serum were generous gifts from Dr. Lee Ann Garrett-Sinha of the University at Buffalo. Data was acquired using Synergy HTX reader (Biotek).

### Statistical analysis

In all graphs, data is presented as mean ± SD of a single experiment. Differences between mean values were determined by ANOVA or Student's *t* test in Prism 7 software (Graph Pad). *P* < 0.05 is considered statistically significant.

## Results

### ST6Gal-1 and α2,6-sialylation in B cell development

**Figure 1:**
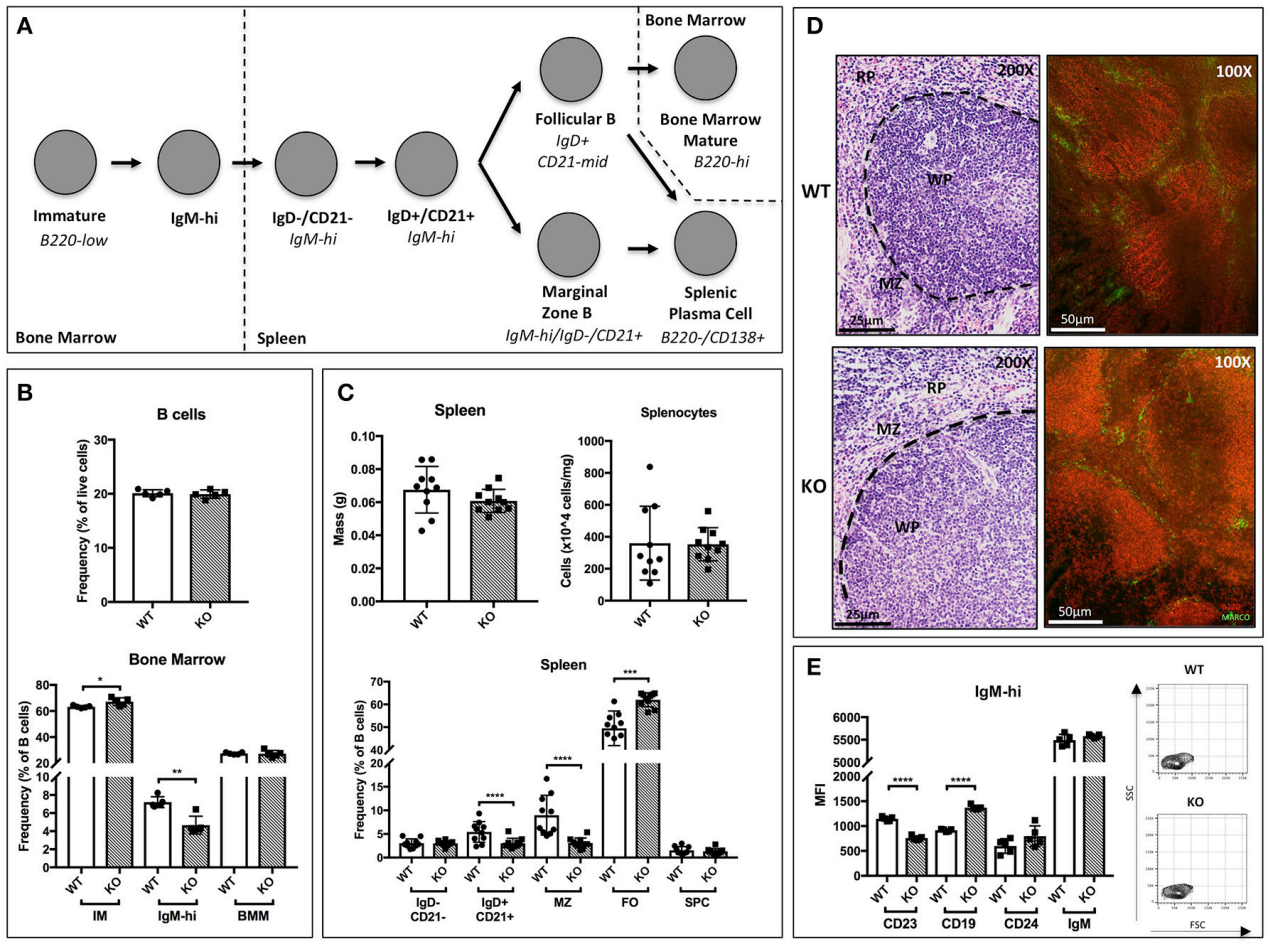
B cell development in St6gal1-KO mice. *St6gal1*-KO (KO) mice were backcrossed onto a C57BL/6J (WT) background for 15 generations to control for strain-specific differences. **(A)** Schematic of B cell development from the immature to mature stages in the bone marrow and spleen, as proposed by Carsetti and colleagues. **(B)** Frequency of B cells in the bone marrow (upper panel) and B cell subpopulations (lower panel) in WT and KO mice (*n* = 5). **(C)** Splenic mass and cell counts in WT and KO mice (upper panels). Frequencies of splenic B cell subpopulations in WT and KO mice (lower panel; *n* = 10). **(D)** Hematoxylin and eosin-stained spleens, with location of relevant anatomical compartments (WP, white pulp; RP, red pulp; MZ, marginal zone). Immunofluorescence microscopy of B220 (red) and marginal zone marker MARCO (green). **(E)** Mean fluorescence intensity of cell surface CD19, CD24, IgM, and CD23 in IgM-high bone marrow B cells, with FSC and SSC of gated cells shown (*n* = 5). *P < 0.05, **P < 0.01, ***P < 0.001, ****P < 0.0001.

The requirement for functional ST6Gal-1 in the development of humoral immunity is well documented (17, 33). However, inconsistencies in the genetic backgrounds of the animals used in previous studies may have introduced genetic changes unrelated to ST6Gal-1 status. Here, we used *St6gal1*-KO mice that have been backcrossed for 15 generations onto the C57BL/6J background. First, we examined how inactivation of ST6Gal-1, resulting in inability to α2,6-sialylate N-glycans, perturbs major B-lineage cell populations in the bone marrow and spleen. Bone marrow B220+ cells were subdivided into B220-low immature fraction, B220-high mature fraction, and an B220-variable/IgM-high fraction, defined elsewhere as transitional (34). Splenic B cells were segregated based on the original gating scheme of Carsetti and colleagues, in which early transitional (T1; IgD-/CD21-/IgM-hi), late transitional (T2; IgD+/CD21+/IgM-hi), marginal zone (IgD-/CD21+/IgM-hi), and follicular (IgD+/CD21-mid/IgM-low/mid) populations are defined (35). In addition, splenic plasma cells (B220-/CD138+) were identified. The developmental scheme associated with these populations is schematically outlined in Figure 1A.

Within the bone marrow, no significant differences in marrow cellularity or frequency of total B220+ cells were observed between C57BL/6J (WT) and *St6gal1*-KO (KO) animals. However, the *St6gal1*-KO marrow contains enlarged immature (IM) and diminished IgM-high populations (Figure 1B) (34). Abundance of recirculating bone marrow mature (BMM) B cells was not significantly different. Alternatively, we used CD24 to separate immature and mature subsets, and resolved IgM-high/CD23- and IgM-low/CD23- transitional and immature B cells. A small CD24+/IgM-high/CD23+ population was also identified, potentially representing late transitional B cells within the marrow (Supplementary Figure 1D) (36). By both schemes, the *St6gal1*-KO marrow had reduced IgM-high B cells, along with a slight increase in immature B cells. Together, these observations suggest a role for ST6Gal-1 in the progression from the immature to earliest transitional B cell stages in the marrow.

In the spleen, no differences were observed between wild-type and *St6gal1*-KO animals in overall organ size or total number of splenocytes. However, the *St6gal1*-KO spleen had reduced IgD+/CD21+ and marginal zone (MZ) B cells, and an expanded follicular (FO) B cell population (Figure 1C). The MZ B cells are maintained by marginal zone precursors (MZP) and type II follicular cells (FO-II) (37). By resolving MZP within the IgD+/CD21+ population, and the FO-I from FO-II cells, we observed that the *St6gal1*-KO mice have reduced MZPs, consistent with defective development from MZP to MZ B cells. The increase in FO B cells was limited to the IgM-low FO-I population, not known to be a MZ precursor (Supplementary Figure 2). Reduced MZ and increased FO B cells in *St6gal1*-KO were confirmed by an alternate gating strategy using CD23 (Supplementary Figure 1B) (38). Histologic examination further supported that the *St6gal1*-KO spleen had reduced co-localization of B220+ (in red) cells with MARCO (in green), a marker of marginal zone macrophages (Figure 1D). Collectively, these observations demonstrate a role for ST6Gal-1 in the development of marginal zone lineage B cells.

Additionally, we noted changes in cell surface expression of specific proteins in the bone marrow IgM-high B cell population. Overall expression of the mature B cell marker CD23 was significantly reduced in *St6gal1*-KO IgM-high B cells. CD23 (FcεRII) is a pro-survival mitogenic receptor induced by B cell activating factor (BAFF) (39, 40). Furthermore, ST6Gal-1 deficient IgM-high cells also exhibited increased expression of CD19 (Figure 1E).

**Figure 2:**
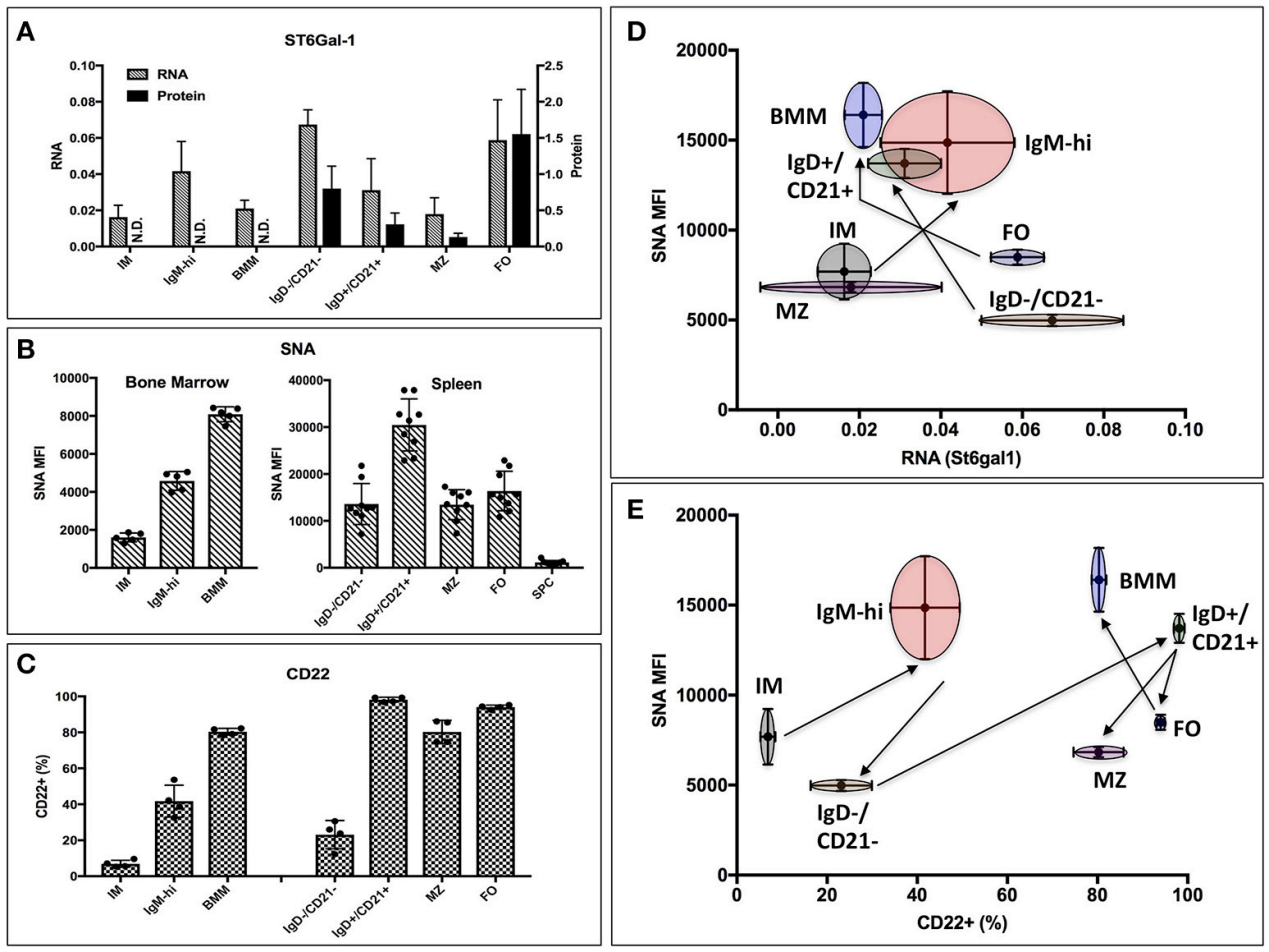
Expression of ST6Gal-1, α2,6-sialyl ligands, and CD22 in B cells. **(A)** Bone marrow immature (IM), IgM-high, and mature (BMM), as well as splenic IgD-/CD21-, IgD+/CD21+, marginal zone (MZ), and follicular (FO) populations were isolated by fluorescence activated cell sorting (FACS) (>94% purity). RT-qPCR was performed for ST6Gal-1 transcripts, and representative results of three independent experiments shown relative to β2-microglobulin (*n* = 3). Western blot analysis of protein levels in splenic populations is quantified relative to β-actin (*n* = 3). **(B)** Mean SNA reactivity is shown for bone marrow and splenic B cell subsets (*n* = 5 or 10). **(C)** Frequency of cell surface CD22 expression in BM and splenic B cell populations (*n* = 5). **(D)** Relative RNA expression of ST6Gal-1 and SNA reactivity are compared, with standard deviations shown in both dimensions, and arrows indicating select developmental steps. **(E)** CD22 expression and SNA reactivity is compared, with standard deviations of measurement shown in both dimensions. Arrows indicate sequence of B cell development.

Thus, global ST6Gal-1 deficiency introduced distinct perturbations at an early transitional stage in marrow and at the follicular/marginal zone decision point in the spleen. To understand if these perturbations corresponded to discrete periods of ST6Gal-1 expression, we quantified endogenous ST6Gal-1 expression, cell surface α2,6-sialyl epitopes, and cell surface CD22, the main B cell lectin that binds α2,6-sialyl ligands, during B cell development in wild-type animals. Endogenous ST6Gal-1 mRNA abundance varied strikingly among immature (IM), IgM-high, and mature B cells (BMM) of the marrow, and the IgD-/CD21-, IgD+/CD21+, marginal zone (MZ), and follicular (FO) B splenocytes (Figure 2A). In the spleen, we observed two prominent maxima, in the IgD-/CD21- early transitional and FO populations, whereas immature (IM), marginal zone (MZ), and bone marrow mature (BMM) stages exhibited minimal ST6Gal-1 expression. ST6Gal-1 protein levels, detected by immunoblot, were generally in agreement with ST6Gal-1 mRNA abundance in the spleen, suggesting efficient translation of St6gal1 transcripts (Figure 2A). In contrast, we observed that cell surface α2,6-sialyl epitope density, revealed using the lectin SNA (*Sambucus nigra* agglutinin), only rarely agreed with endogenous ST6Gal-1 expression in stage-by-stage comparisons (Figure 2B). IgD+/CD21+ B cells had the highest SNA reactivity (MFI > 30,000) but unremarkable endogenous ST6Gal-1 expression on both the protein and mRNA levels. On the other hand, FO B cells were strikingly enriched for ST6Gal-1 but exhibited minimal cell surface α2,6-sialyl epitopes (Figures 2A,B). Most populations in the *St6gal1*-KO animal were SNA-negative, confirming the primacy of ST6Gal-1 in the generation of α2,6-sialyl structures in the B lineage. Curiously, the *St6gal1*-KO MZ B cells had a slight but distinct SNA signal of ~1000, which was ~5% of the wild-type MZ counterparts, but at least 4-fold higher than other B cell populations (Supplementary Figure 3). This was likely due to ST6GalNAc sialyltransferases, as reported by others, and was not explored further here (41). Cell surface expression of CD22 is shown in Figure 2C. IgD+/CD21+ and mature (MZ/FO) B cells were uniformly high in CD22 expression; a significant body of literature exists on the role of CD22 as an accessory modifier of BCR signaling in these populations. We also observed moderate cell surface CD22 in the marrow IgM-high and splenic IgD-/CD21- early transitional populations. Overall, cell surface α2,6-sialyl structures paralleled CD22 expression to a greater degree than it did endogenous ST6Gal-1 expression (Figures 2D,E). Furthermore, although stages with highest ST6Gal-1 expression (IgD-/CD21-, FO) often preceded stages with abundant α2,6-sialyl structures (IgD+/CD21+, BMM), this was not the case for the bone marrow IgM-high stage, which exhibited high SNA reactivity despite developing from a precursor (IM) with minimal ST6Gal-1 expression (Figure 2D, arrows).

Taken together, the data indicate not only the need for ST6Gal-1 mediated sialylation during B lymphopoiesis, but also suggests that stage-specific regulation of endogenously expressed ST6Gal-1 cannot fully account for the cognate α2,6-sialyl ligands in B cell development.

### Cell-autonomous and cell non-autonomous ST6Gal-1 in B cell development

Significant levels of ST6Gal-1 exist in extracellular spaces, particularly the systemic circulation. Extracellular, or extrinsic ST6Gal-1, contributes to the sialylation of mature and progenitor myeloid-lineage cells and the F_c_ region of circulating IgG (14, 25, 27, 28). By extrinsic sialylation, ST6Gal-1 impedes granulocyte production and suppresses inflammation (26).

**Figure 3:**
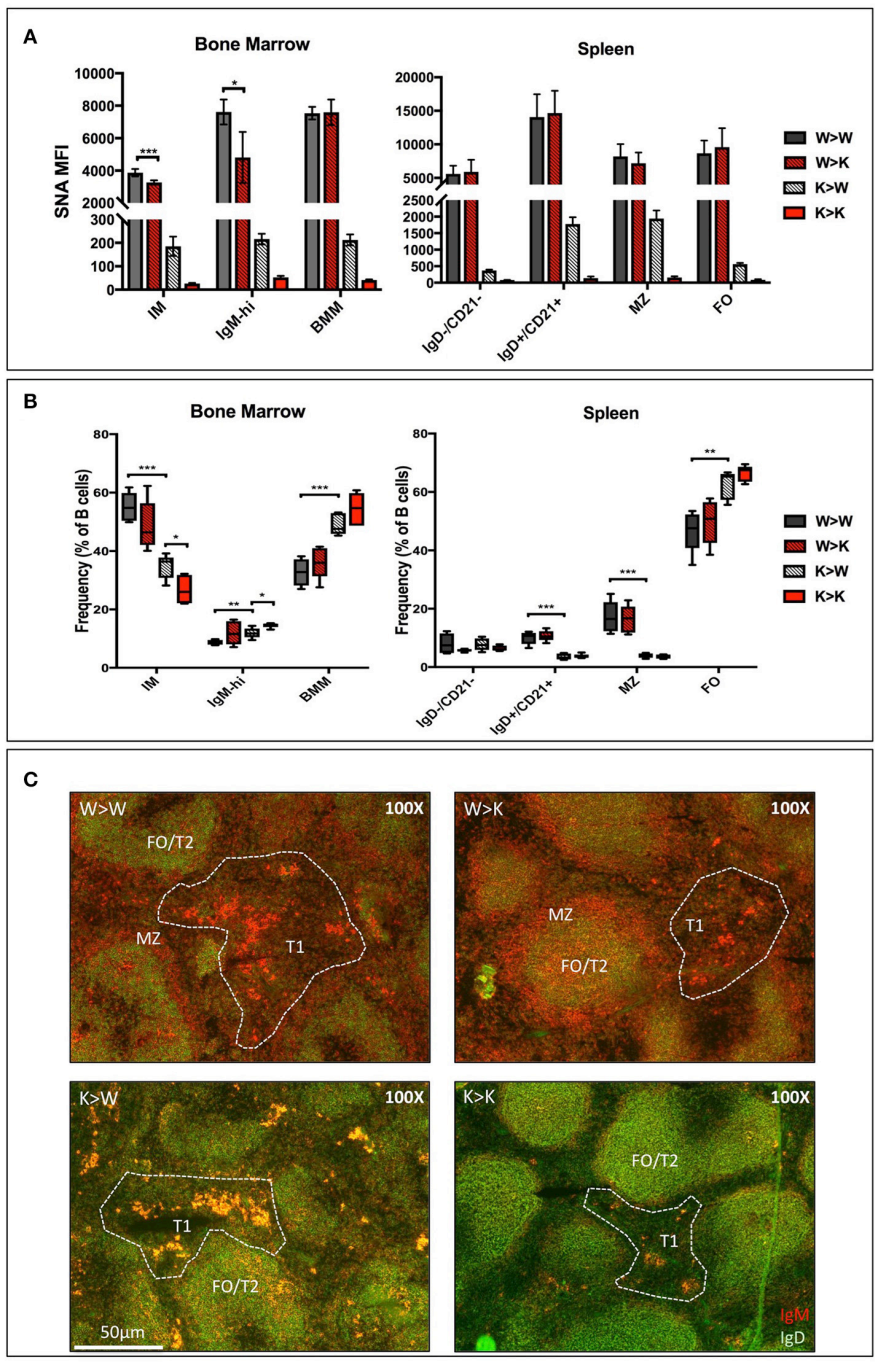
Cell non-autonomous ST6Gal-1 influences sialylation and abundance of early transitional B cell populations. CD45.1+ whole bone marrow cells from wild-type or St6gal1-KO mice were adoptively transferred to irradiated CD45.2+ hosts. Mice were allowed to recover for 6 weeks before analysis of bone marrow and splenic B cells. **(A)** SNA reactivity of bone marrow and splenic B cell subsets of CD45.1+ donor cells. **(B)** Frequencies of CD45.1+ IM, IgM-high, BMM, IgD-/CD21-, IgD+/CD21+, MZ, and FO B cells as a fraction of total CD45.1+ B cells (*n* = 5). **(C)** Immunofluorescence microscopy staining anti-IgM (red) and anti-IgD (green) in chimeras. Splenic B cell populations indicated are identified accordingly - T1: IgM+/IgD-, extrafollicular; T2 and FO: IgM-variable/IgD+, follicular, MZ: IgM+/IgD-, marginal sinus. *P < 0.05, **P < 0.01, ***P < 0.001, ****P < 0.0001.

In order to distinguish between the contributions of the ST6Gal-1 intrinsically expressed in B cells and ST6Gal-1 of extrinsic origin, CD45.1+ wild-type or *St6gal1*-KO donors were used to reconstitute the hematopoietic compartments of irradiated CD45.2+ wild-type or *St6gal1*-KO recipients. First, we assessed the cell surface α2,6-sialylation of B lineage populations using SNA (Figure 3A). Wild-type (ST6Gal-1 competent) B cells generally maintained a similarly high degree of cell surface α2,6-sialylation regardless of the ST6Gal-1 status of the hosts, highlighting the importance of cell-autonomous α2,6-sialylation in B cells. A notable exception was the marrow immature and IgM-high fractions, where wild-type cells had reduced cell surface SNA reactivity (15–37% diminished) when repopulating ST6Gal-1-null recipients, suggesting an uncompensated requirement for cell non-autonomous ST6Gal-1 in the maintenance of cell surface sialylation of these populations. In the absence of endogenous ST6Gal-1 expression, sialylation by cell non-autonomous ST6Gal-1 also occurred in all other B lineage populations examined; *St6gal1*-KO B cells, which could not self-sialylate, had significantly reduced SNA-reactivity when propagated in *St6gal1*-KO recipients, when compared to wild-type recipients.

The relative distribution of donor-derived B cells 6 weeks after transplantation is summarized in Figure 3B. We also stained for IgM and IgD expressing splenic B cells to discriminate between migrating extrafollicular T1, follicular T2/FO, and marginal zone MZ B cells, as described elsewhere (Figure 3C) (38). Generally, reconstitution with *St6gal1*-KO B cells manifested as a reduced MZ and IgD+/CD21+, but increased FO compartment, regardless of the ST6Gal-1 status of the recipients, paralleling the observations in the global *St6gal1*-KO mouse (see Figure 1C). The increase in FO B cells was accompanied by an increase in their recirculating variant, the bone marrow mature (BMM) population. In contrast, absence of systemic ST6Gal-1 (in *St6gal1*-KO recipients) led to a diminished IgD-/CD21- compartment. This reduction, dependent on host expression of ST6Gal-1, was quantitatively significant in comparisons of male, but not female mice (Supplementary Figure 4).

**Figure 4:**
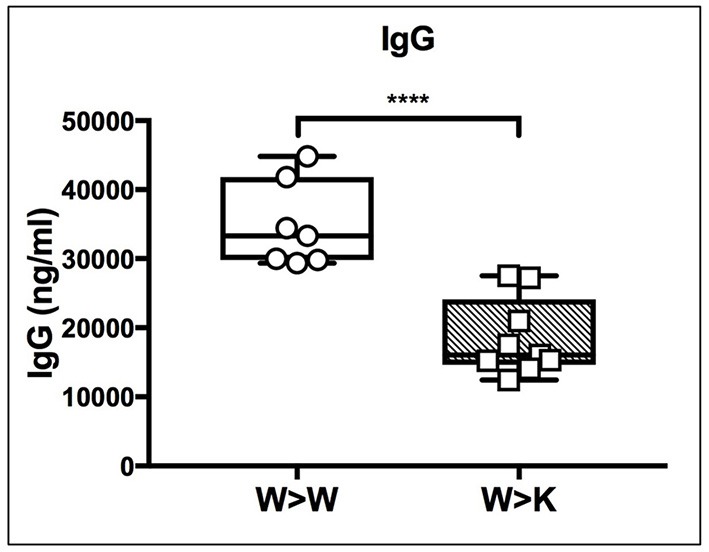
Cell non-autonomous ST6Gal-1 influences serum IgG. Wild-type or St6gal1-KO mice reconstituted for 6 weeks with wild-type bone marrow were assayed for serum IgG (*n* = 7, 9). **** *P* < 0.0001.

Thus, the loss of B cell-autonomous ST6Gal-1 led to a clear reduction in the MZ lineage cells. On the other hand, the loss of cell non-autonomous ST6Gal-1 resulted in reduced IgD-/CD21- splenic early transitional B cells. These results were validated by microscopy showing reduced IgM-high T1 populations in *St6gal1*-KO recipients reconstituted by either wild-type or *St6gal1*-KO cells (W>K and K>K, respectively in Figure 3C). This reduced T1 population could not be compensated by B cell intrinsic expression of ST6Gal-1. To understand if host ST6Gal-1 status affected antibody-producing function of the B cell compartment, we quantified titers of serum immunoglobulin G (IgG) in chimeras in which wild-type bone marrow was used to reconstitute wild-type or *St6gal1*-KO mice. We noted a striking decrease in circulatory IgG in hosts lacking ST6Gal-1, confirming the functional importance of systemic ST6Gal-1 in maintenance of antibody production (Figure 4).

**Figure 5:**
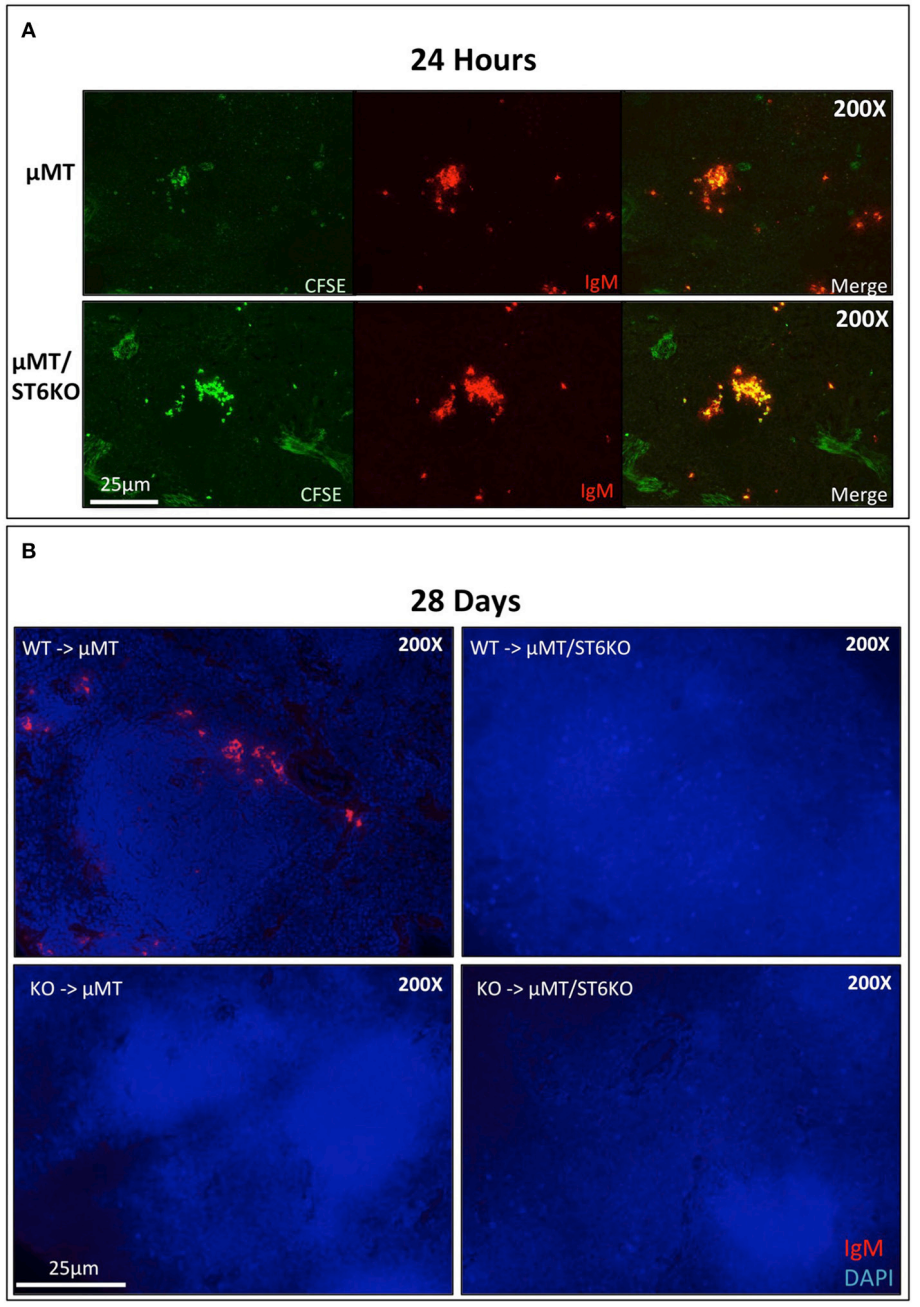
Systemic ST6Gal-1 influences long-term survival, but not homing of splenic B cells. **(A)** CFSE-labeled splenocytes from day 6 wild-type mice were intravenously injected into μMT and μMT/ST6KO mice. 24 h later, spleens were analyzed. CFSE+ IgM+ cells were identified in spleens of recipient mice 24 hrs post-injection. The frequency of adoptively transferred B cells was equal between μMT and μMT/ST6 DKO mice (quantified in Supplementary Figure 5C). **(B)** CD3−/B220+ wild-type or *St6gal1*-KO splenic B cells were transferred intravenously into μMT or μMT/ST6KO mice, and recipients sacrificed after 28 days. Representative microscopic analysis of spleens for IgM expression is shown (*n* = 3).

Our results show that cell non-autonomous ST6Gal-1 influences sialylation of marrow immature and IgM-high B cells and population size of splenic IgD-/CD21- cells. There are several possible explanations for the reduced early transitional population in ST6Gal-1 deficient hosts. First, we considered diminished migration of the early transitional B cells to the spleen. CFSE-labeled splenocytes were intravenously infused into B cell-deficient (μMT) mice that either express or lack ST6Gal-1. The donor splenocytes, from post-natal day 6 wild-type mice, lacked B cells beyond the IgM-high transitional stages (Supplementary Figure 5A). 24 h post-transfer, splenic CFSE+ B cells localized near arteriolar structures (Supplementary Figure 5B), but the recipient ST6Gal-1 status did not alter the ability of transitional B cells to migrate (Figure 5A, Figure S5C). Another possibility is that systemic ST6Gal-1 enhances transitional B cell survival or development by the extrinsic pathway of sialylation. This hypothesis is supported by the observed changes in α2,6-sialylation on marrow B cell populations in the absence of recipient ST6Gal-1 expression (Figure 3A). To test the survival of B cells in the presence or absence of extrinsic ST6Gal-1, we adoptively transferred wild-type or *St6gal1*-KO CD3-/B220+ splenic B cells into μMT mice that either express or lack ST6Gal-1. After 28 days, recipients were sacrificed and spleens analyzed for IgM expression, which is absent on the μMT background. Strikingly, we observed IgM+ B cells only when both transferred B cells and recipients expressed ST6Gal-1 (Figure 5B). Collectively, our adoptive transfer experiments suggest a role for extrinsic ST6Gal-1 in long-term survival, but not in homing, of B cells.

**Figure 6:**
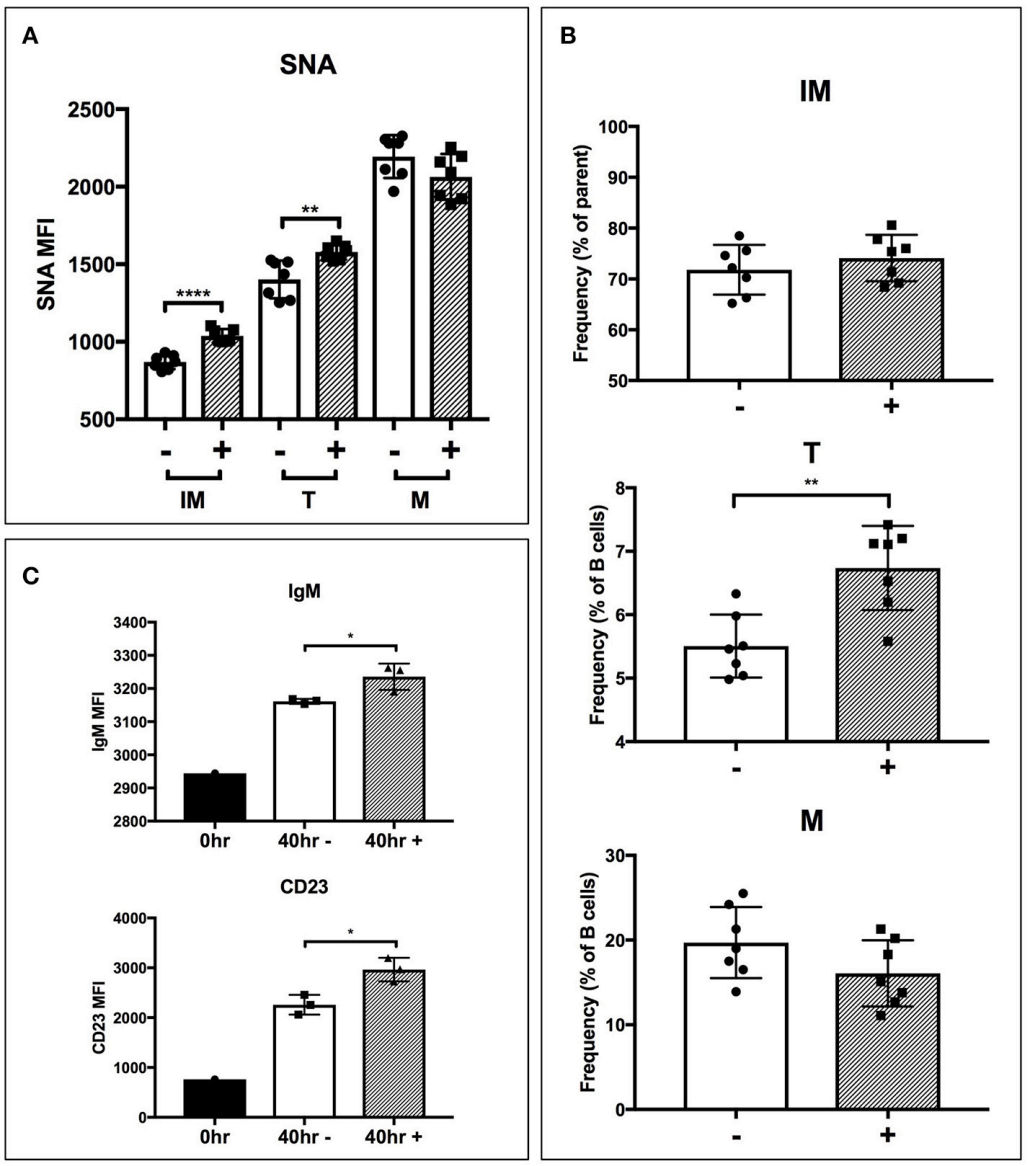
Extrinsic sialylation promotes transitional B cell development and CD23 expression. Wild-type immature B cells were cultured for 40 h with or without recombinant ST6Gal-1. Phenotypically B220-low (IM), IgM-hi (T), and B220-high (M) B cells are designated. In all panels, absence or presence of recombinant ST6Gal-1 (rST6G) in culture is indicated with “−” or “+.” **(A)** Mean fluorescence intensity for SNA is shown (*n* = 7). **(B)** Relative size of B cell populations after culture period (*n* = 7). **(C)** Expression of cell surface IgM and CD23 in the transitional population before culture, and after culture with or without extrinsic sialylation (*n* = 3). Results are representative of 3 independent experiments. *P < 0.05, **P < 0.01, ****P < 0.0001.

To test if extrinsic ST6Gal-1 affects transitional B cell development, immature B cells (IgM-/B220+) were obtained by magnetic cell sorting and cultured for 40 hrs with ST6Gal-1 or vehicle control. Control cultures increased CD23 (FcεRII) and B220 expression, and downregulated CD24, as expected during development (Supplementary Figures 6A,B). There was a statistically significant increase in SNA reactivity in ST6Gal-1-treated cultures in the immature and transitional populations (Figure 6A), as well as a slightly increased transitional population (Figure 6B). Notably, transitional B cells in the extrinsically treated cultures also expressed more CD23 and IgM (Figure 6C). Together, these results are highly consistent with *in vivo* data from bone marrow chimeras, and support the notion that extrinsic ST6Gal-1 enhances the development of transitional B cells from the immature stage.

### ST6Gal-1 extrinsic sialylation and pro-survival signaling

**Figure 7:**
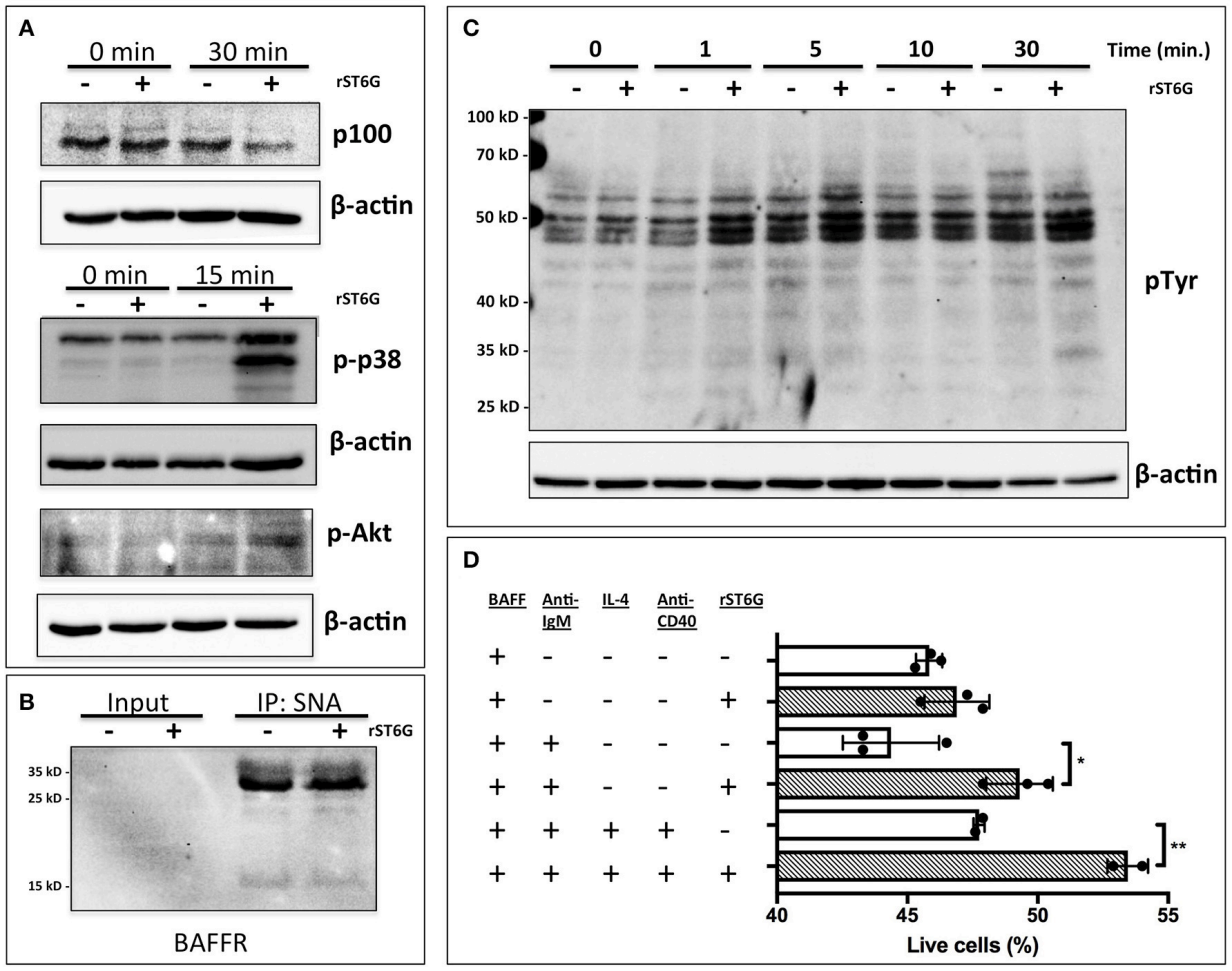
Extrinsic sialylation enhances BAFF and BCR-mediated pro-survival signaling. **(A)** Wild-type bone marrow immature B cells were extrinsically sialylated with recombinant enzyme (rST6G), then stimulated with recombinant BAFF for 15 or 30 min, before western blot analysis of cytosolic proteins. **(B)** The membrane portion of cell lysate was subjected to immunoprecipitation with SNA-agarose beads, and the enriched fraction probed for BAFFR by western blot. ~5% of input is shown, whereas ~20% of input is represented in the immunoprecipitation. **(C)** Bone marrow CD23-/B220+ B cells were purified by magnetic separation, extrinsically sialylated by recombinant ST6Gal-1, then stimulated with functional grade anti-IgM F(ab')2 for indicated times. Anti-pTyr (4G10) blot identifies total tyrosine phosphorylation events. **(D)** Immature B cells were cultured in presence of BAFF and ST6Gal-1 and stimulated with anti-IgM F(ab')2 antibody with or without IL-4 and anti-CD40 antibody. Survival was quantified after 18 h by DAPI uptake, and results are representative of 3 independent experiments (*n* = 3). *P < 0.05, **P < 0.01.

A number of factors, including B cell activating factor (BAFF), prolactin, and Toll-like receptor 7 (TLR7) agonists, augment the development and survival of early transitional B cells (42–44). BAFF, a cytokine with sex-dependent variation, is necessary for early transitional B cell development and CD23 expression (45, 46). In order to gain mechanistic insight into how extrinsic ST6Gal-1 affects transitional B cell development, immature B cells were extrinsically sialylated *ex vivo* with recombinant ST6Gal-1 (rST6G) for 2 h, then stimulated with murine BAFF. rST6G exposure resulted in increased degradation of cytosolic p100, a mediator of non-canonical NF-kB signaling, and robust p38 phosphorylation. There was also a modest elevation of AKT phosphorylation by rST6G (Figure 7A). Each of these pathways can convey anti-apoptotic signals downstream of the BAFF receptor (BAFFR) (47–49). ST6Gal-1-dependent sialylation of the TNF-α receptor has been shown to enhance pro-survival signaling in cancer cells (50). Therefore we considered the possibility that BAFFR, a member of the TNFR superfamily with at least one N-glycosylation site, is a direct target of extrinsic ST6Gal-1. However, when SNA-reactive membrane proteins were immunoprecipitated, neither the 17 kD monomer nor 34 kD dimer of BAFFR were enriched after rST6G treatment, indicating that BAFFR was not sialylated to an appreciable extent by extrinsic ST6Gal-1 (Figure 7B). Furthermore, cell surface retention of BAFFR was not altered by rST6G treatment (data not shown).

Tonic, low-level B cell receptor activation is obligatory for BAFFR signaling (42). Moreover, CD22, a siglec that recognizes the α2,6-sialyl structures synthesized by ST6Gal-1, is a well-known regulator of B cell receptor (BCR) signaling. We next hypothesized that extrinsic sialylation enhances BAFF signaling by modulation of BCR activation. To test this, we extrinsically sialylated bone marrow immature and transitional B cells prior to BCR stimulation *in vitro*, and observed that the rST6G-treated B cells exhibited increased tyrosine phosphorylation as soon as 1 min after stimulation (Figure 7C).

As targets of negative selection, early transitional B cells are central to the development of autoimmunity (5). Since we observed activation of anti-apoptotic pathways in this population after extrinsic sialylation with rST6G, we hypothesized that extrinsic sialylation would enable transitional B cells to resist apoptosis by negative selection. We stimulated sialylated immature and transitional B cells with B cell receptor activation, with or without T cell help signals, in the presence of murine BAFF. ST6Gal-1 enhanced survival under conditions of negative selection, with or without T cell help (Figure 7D). Consistent with this mechanism, significant reductions of autoantibodies against histones as well as dsDNA were observed in the blood of ST6Gal-1deficient compared to wild-type mice (Supplementary Figure 7A). We observed recapitulation of reduced anti-dsDNA antibodies, in bone marrow chimeras lacking host ST6Gal-1 and repopulated with wild-type cells (Supplementary Figure 7B). Together, these results support a role for extrinsic ST6Gal-1 as a pro-survival factor in transitional B cell development, which may also explain why transfused splenic B cells, while competent in trafficking, were unable to persist in the spleen after 28 days in ST6Gal-1 deficient hosts (Figure 5B).

## Discussion

The inability to generate α2,6-sialyl glycans due to deficiency of the sialyltransferase ST6Gal-1 results in a plethora of humoral defects, including an attenuated response to antigenic challenges, impaired B cell proliferation, and reduced serum IgM (17). However, it is unclear whether the biochemical origin of the α2,6-epitope is important to these processes. ST6Gal-1 is expressed natively in both B lineage and non-B lineage cells. In addition, a considerable pool of extracellular ST6Gal-1 is present in systemic circulation. We have previously demonstrated a role for extracellular ST6Gal-1 in managing inflammation by inhibiting the production of inflammatory cells (15, 16, 26). In this report, we used the global ST6Gal-1 null mouse, hematopoietic chimeras generated by adoptive transfer, and *ex vivo* studies to show that contextual differences in the origin of ST6Gal-1 are a significant determinant of B cell development and function.

The humoral defects in ST6Gal-1 deficient animals are attributed to disrupted engagement and signaling of CD22, and potentially Siglec-10/G (51). Both CD22 and Siglec-10/G are ITIM-containing receptors of the siglec family of lectins, and recognize the α2,6-sialyl epitope created by ST6Gal-1 (52). Although less is known about Siglec-10 signaling, CD22 regulates B lymphocyte function by both ligand-dependent and -independent mechanisms (53), and can be either a positive or negative regulator of BCR signaling (51, 54, 55). Indeed, CD22 deficiency results in hyper-responsiveness to BCR stimulation, but ST6Gal-1 deficient B cells paradoxically exhibit a muted response (55). Our data are in line with the idea that B cell native ST6Gal-1 is important for CD22-mediated suppression of BCR signaling, putatively by elaboration of *cis*-interacting CD22 ligands. In contrast, antigen presentation by ST6Gal-1 expressing non-B cells can recruit CD22 and Siglec-10/G to the immunological synapse by *trans* interactions, suppressing BCR signaling and enforcing apoptosis (56–58). This toleragenic regulation by CD22 and Siglec-G/10 has been proposed as a mechanism for host discrimination between self and non-self (59), safeguarding against the development of autoimmunity (60).

The differential contributions of B cell-autonomous and non-B cell-autonomous ST6Gal-1 were examined using bone marrow chimeras between *St6gal1*-KO and wild-type mice. Our data show an absolute requirement for B cell-autonomous ST6Gal-1 in marginal zone B cell lineage development; *St6gal1*-KO precursors, regardless of recipient ST6Gal-1 status, were unable to re-establish splenic late transitional and marginal zone B cell compartments (see Figure 3B). The marginal zone B cell population, a mature non-recirculating subset occupying the marginal sinus, is key to T-independent B cell responses and scavenging of blood borne immune complexes. In contrast, hematopoietic progenitors, regardless of intrinsic ST6Gal-1 expression, failed to fully re-establish the splenic IgD-/CD21- population in *St6gal1*-KO recipients, a population subject to negative selection by antigenic engagement, highlighting a role for B cell non-autonomous ST6Gal-1 in B lymphopoiesis at the early transitional stage (see Figure 3B). ST6Gal-1 sufficient B cells were able to home but could not persist in *St6gal1*-KO spleens (see Figure 5A, Supplementary Figure 5), and bone marrow chimeras lacking systemic ST6Gal-1 had reduced total blood IgG (see Figure 4). Together, these observations indicate non-redundant roles for B cell-dependent and B cell-independent ST6Gal-1 in B cell development and function.

B cell non-autonomous ST6Gal-1 can influence B cell development through the intrinsic generation of glycans on antigen-presenting cells or extrinsic remodeling of B cell surface glycans by the extracellular ST6Gal-1 pool. Extracellular ST6Gal-1, dynamically upregulated by the liver in response to inflammation, can systemically modulate glycan-dependent processes by generating α2,6-sialic acid on both central and peripheral immune cells (14, 25, 26). Triggered by inflammatory and thrombotic stimuli, extrinsic sialylation by ST6Gal-1 responds to changing physiologic cues to curb inflammatory processes. Our observations suggest that this mechanism is at least partially responsible for the functional alterations associated with the loss of non-B cell ST6Gal-1. First, at specific stages of B cell development, cell surface α2,6-sialyl glycans were not always concordant with the level of endogenous ST6Gal-1 expression (see Figure 2). The discordance was especially notable in the IgM-high B cells of the marrow, which also exhibited a reduction in α2,6-sialyl glycans in chimeras lacking host ST6Gal-1 expression. Moreover, *St6gal1*-KO hematopoietic cells, incapable of autonomously synthesizing α2,6-sialyl structures, nevertheless gained considerable α2,6-sialic acid in the B lineage when transplanted into wild-type recipients (see Figure 3A). Finally, extrinsic sialylation in the B lineage was recapitulated *in vitro* using recombinant ST6Gal-1. When added to isolated immature B220+ marrow cells, rST6G sialylated immature and transitional subsets, enhanced cell surface IgM and CD23 expression (see Figure 6C) and enhanced pro-survival signaling pathways (see Figure 7A). Sialylation of B cells with rST6G was sufficient to enhance their survival under conditions of antigen engagement, with or without T cell help (Figure 7D). The effects of extrinsic sialylation in this instance may parallel the acquisition of immune competence, characterized by resistance to BCR-induced apoptosis, sensitivity to T cell help signals, and greater stability of the BCR signaling complex that occurs at the T1/T2 transition (61).

Transitional B cells represent a heterogeneous population of immature B cells in the process of acquiring the hallmarks of maturity. The molecular and cellular requirements enabling this bridging population to survive as it migrates into splenic follicles and attains functional maturity are useful biological chokepoints in the enforcement of self-tolerance. The mechanisms maintaining tolerance include cell death by negative selection, clonal deletion by receptor editing, and anergy (62). The minority of cells that successfully enter the long-lived mature B cell pool are characterized by a productive response to antigenic stimulation, dependence on BAFF, upregulation of IgD and CD23, and a tonic level of BCR signaling that permits continued survival (63). In this report, we have followed the original descriptions of transitional B cells by Carsetti and colleagues, delineating several IgM-high populations in the bone marrow and spleen, differentiated by expression of IgD, CD21, and CD23 (5, 35). Since these earlier publications, however, significant work has advanced the understanding of transitional B cell development and function. In a refinement of the developmental scheme we have presented (Figure 1A), David Allman and colleagues demonstrated that immature B cell subsets in the spleen are marked by their expression of the C1q receptor homolog AA4/CD93, and can be further resolved into three separate populations (T1, T2, T3) based on CD23 and IgM (64). Recent reports suggest that the newly-defined T3 population represents an anergic B cell population with a role in tolerance, but its exact position in B cell development remains incompletely understood (65). Hence the T3 population was not included in our analysis. Furthermore, the T2 population, as originally defined by Loder et al. (35), has been subsequently shown by Allman's group to contain a large reservoir of marginal zone precursors (66). Moreover, this T2 population was interpreted to be the target of positive selection into the mature B cell pool due to evidence of a proliferative burst. An alternative model, wherein all CD93+ transitional subsets are non-cycling, and T2 cells represent a follicular subset in the process of differentiation into marginal zone B cells, has also gained significant traction (67). Finally, the processes of selection may not be limited to specific microenvironmental niches, as indicated by the presence of T2-like CD23+ late transitional B cells, receptive to BAFF, in the bone marrow (36). In our study, we note a significant reduction of these CD24+/IgM-hi/CD23+ B cells in mice with ST6Gal-1 insufficiency (Supplementary Figure 1D). Thus, B cell development to the IgD+ stage may proceed in parallel in the bone marrow and spleen. Regardless of evolving differences in the methods of gating, our findings are consistent with the involvement of extrinsic sialylation in receptivity to BAFF signaling.

Early transitional B cells, the physiologic targets of negative selection, are a labile population with the potential to harbor autoreactive clones. In addition to intrinsic developmental cues, niche-based extrinsic factors are already known to influence B cell development and negative selection (68). One major factor, BAFF, synergizes with BCR signaling to promote survival and differentiation through these early peripheral stages, greatly influencing the mature immune repertoire (69). Several observations suggest BAFF mediates the developmental effects of non-B cell ST6Gal-1. The bone marrow transplantation data showed a tendency toward male-specific *in vivo* differences in the IgD-/CD21- population upon loss of host ST6Gal-1, consistent with reported sex-associated variation in serum BAFF and the emerging role of sex hormones in the regulation of BAFF expression (46, 70, 71). ST6Gal-1-associated perturbations in early transitional B cells were observed in both bone marrow and splenic transitional compartments, consistent with the multi-stage significance of BAFF for immature B cells (72, 73). Alterations in expression of CD23 in global *St6gal1-KO* mice, as well as in B cells treated with rST6G *ex vivo*, also suggest a BAFF-dependent mechanism (40). Finally, BAFF signaling was augmented in immature B cells pre-treated with extrinsic ST6Gal-1 (see Figure 7A). Serum BAFF levels are highly associated with autoimmune disease severity in both humans and rodent models (74–76). The mechanistic basis of this is thought to be through Transmembrane activator and CAML interactor (TACI) and B cell maturation antigen (BCMA), receptors for BAFF implicated in the survival of long-lived plasma cells (77). BAFFR, the highest affinity receptor for BAFF, is critical for peripheral B cell development and is thought to be preferentially expressed on non-autoreactive cells (42). In our autoantibody analysis, though we noted striking differences in autoantibody titers between wild-type and global ST6Gal-1 KO mice, autoantibody titers in bone marrow chimeras in which wild-type donor cells were reconstituted in wild-type or ST6Gal-1 deficient hosts were largely similar (Supplementary Figure 7). These assays largely utilized pure recombinant protein, but in some instances they were limited by the use of DNA as an antigen. Furthermore, these measurements were made only 6 weeks post-transplantation, which may be insufficient for the regeneration of a complete immune repertoire. However, ST6Gal-1 deficient hosts nevertheless exhibited a significant reduction in total IgG, indicating the importance of B cell non-autonomous ST6Gal-1 in humoral immunity. In light of our findings, a potential hypothesis is that systemic ST6Gal-1 acts as a licensing factor that allows non-autoreactive transitional B cells to utilize BAFF, in line with the well-established tolerogenic role for sialic acid.

Taken together, our data outline different roles for the ST6Gal-1 natively expressed in B-lineage cells, non-B lineage cells, and the pool of systemic ST6Gal-1. In view of the newer understanding of the biochemical feasibility and biological importance of the pool of extracellular ST6Gal-1, our observations define a role for circulatory ST6Gal-1 as a systemic extrinsic factor guiding the development and maintenance of humoral immunity, with therapeutic potential as a vaccine adjuvant.

## Author contributions

EI conceived, designed and performed experiments, and wrote manuscript. JL provided critical oversight, conceived and designed research, and wrote manuscript.

### Conflict of interest statement

The authors declare that the research was conducted in the absence of any commercial or financial relationships that could be construed as a potential conflict of interest.
