# Supplementary material for: Systemic ST6Gal-1 Is a Pro-survival Factor for Murine Transitional B Cells
Source: Front Immunol. 2018 Sep 20;9:2150. doi: 10.3389/fimmu.2018.02150 (PMC6159744; doi:10.3389/fimmu.2018.02150)
Supplement: Supplementary file 1 [file Data_Sheet_1.docx]

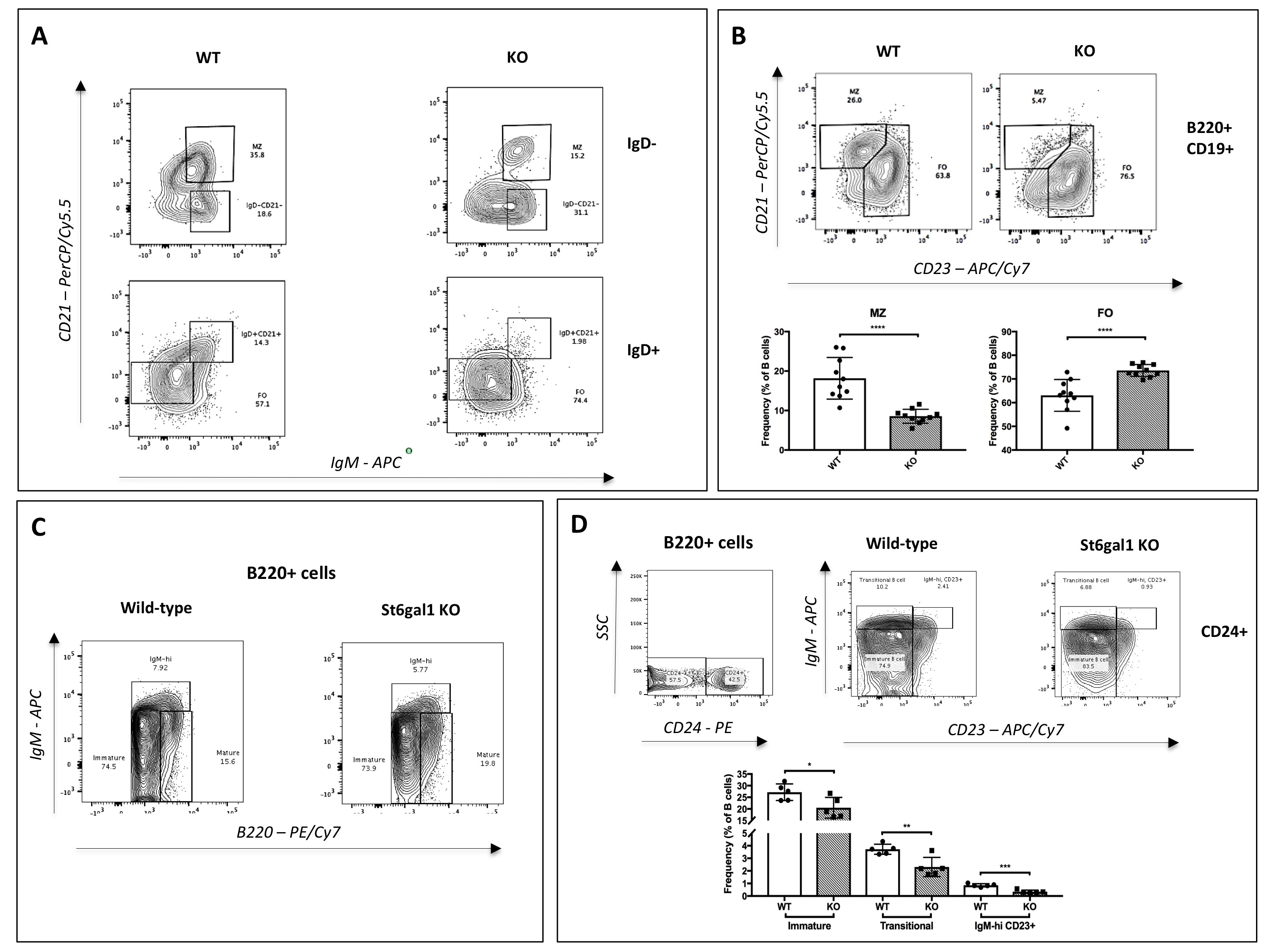


**Supplementary Figure 1. Representative gating for splenic and bone marrow flow cytometry.** (A) Splenic B cell (B220+/CD19+) populations were identified using IgD, IgM, and CD21. IgD-/CD21- (B220+/CD19+/IgD-/CD21-/IgM+), MZ (B220+/CD19+/IgD-/CD21+/IgM+), IgD+/CD21+ (B220+/CD19+/IgD+/CD21+/IgM+), and FO (B220+/CD19+/IgD+ /CD21-/IgM-low/mid). (B) Alternative gating scheme for splenic B cells utilizing CD23 and CD21 to distinguish marginal zone and follicular lineage cells was used. Frequency of marginal zone (B220+/CD19+/CD21+/CD23-low) and follicular (B220+/CD19+/CD21-low/mid/CD23+) B cells in wild-type and St6gal1-KO mice. (C) Gating strategy for bone marrow B220+ B cells using B220 and IgM. Immature (B220-low/IgM-low), IgM-high (B220-variable/IgM-high), and bone marrow mature (B220-high/IgM-low) B cells are shown. (D) Alternative gating scheme for bone marrow B cells utilizing heat-specific antigen (HSA)/CD24, CD23, and IgM. B220+ cells were gated on CD24, and the CD24+ immature fraction was resolved into IgM-mid/low immature B cells, IgM-high transitional B cells, and IgM-high CD23+ B cells. Frequencies of each population is shown in graphical format. * P < 0.05, ** *P* < 0.01, *** P < 0.001, **** *P* < 0.0001.


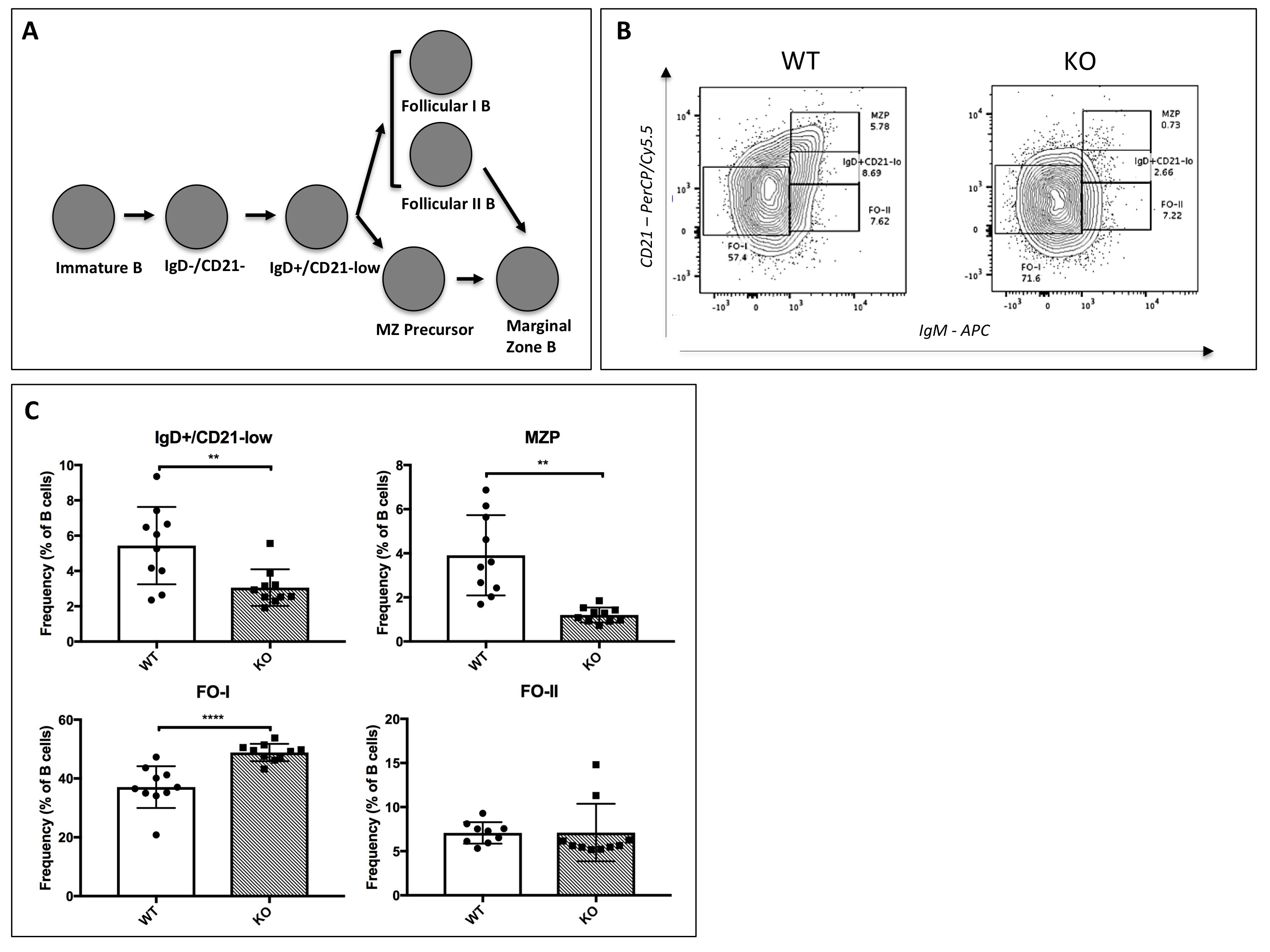


**Supplementary Figure 2. ST6Gal-1 deficiency reduces marginal zone precursors but not follicular type II B cells.** (A) Schematic of splenic B cell maturation, showing that both marginal zone precursor cells and follicular type II B cells can develop into marginal zone B cells and thus be responsible for their deficiency. (B) Representative flow cytometry of gating scheme of IgD+ B cells in wild-type and *St6gal1*-KO spleens, including MZP (B220+/CD19+/IgD+/IgM+/CD21-hi), IgD+/CD21-low (B220+/CD19+/IgD+/IgM+/CD21-low), FO-I (B220+/CD19+/IgD+/IgM-low/neg/CD21-low/neg), and FO-II (B220+/CD19+/IgD+/IgM+/CD21-) B cells. (C) Splenic IgD+/CD21-low and MZP B cells were deficient in the St6gal1-KO mouse, and FO-I, but not FO-II B cells were increased (n=10). These results argue in favor of a deficiency in maturation along the MZP -> MZ lineage, rather than FO-II -> MZ route. The relative increase in classical FO-I follicular B cells suggests a compensatory increase in maturation down the follicular lineage in the absence of marginal zone development. ** *P* < 0.01, **** *P* < 0.0001.


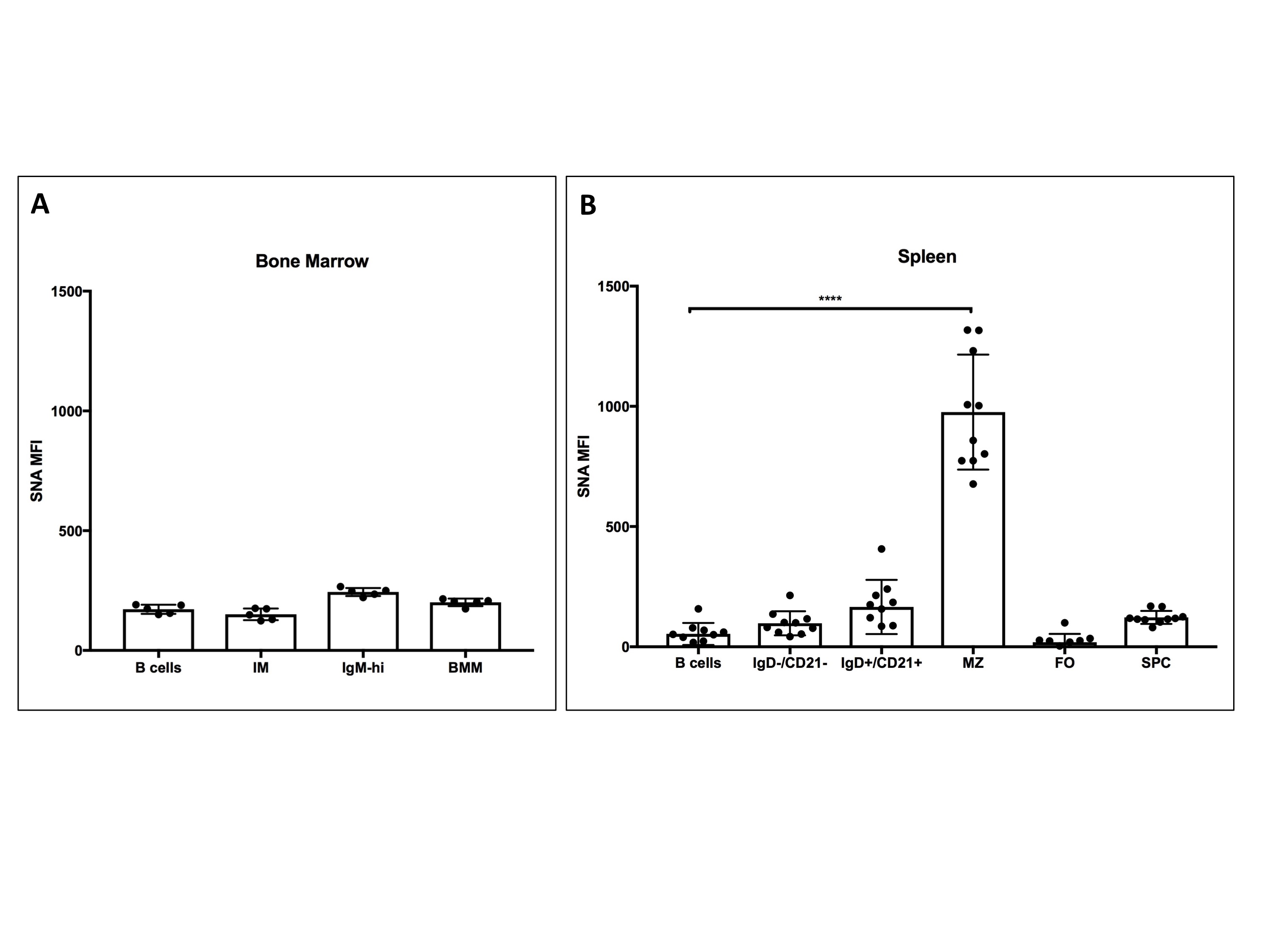


**Supplementary Figure 3. St6gal1-KO marginal zone B cells retain SNA reactivity.** Mean fluorescence intensity for SNA is shown for total B cells and B cell subpopulations in (A) the bone marrow and (B) spleen of *St6gal1*-KO mice. The values shown are negligible in stage-by-stage comparison with wild-type mice, indicating that ST6Gal-1 is responsible for the vast majority of SNA-reactive α2,6-linkages in the B cell lineage. However, a distinct SNA-signal is present in the marginal zone B cell lineage even in the absence of ST6Gal-1 expression. **** *P* < 0.0001.

**
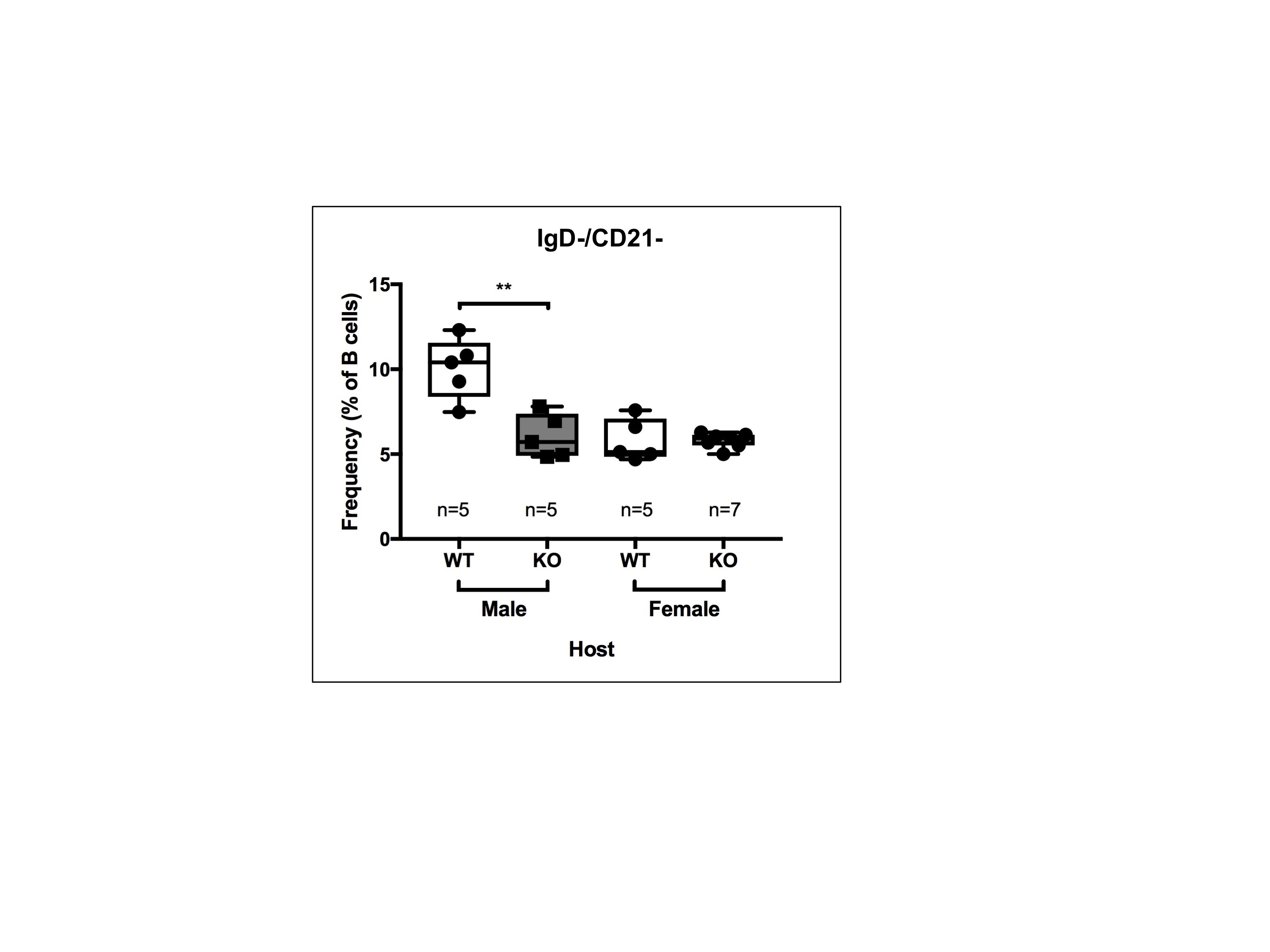
**

**Supplementary Figure 4. Splenic IgD-/CD21- B cells by host genotype and sex.** Wild-type and St6gal1-KO bone marrow was adoptively transferred into irradiated wild-type and St6gal1-KO recipients. Through use of the CD45.1/CD45.2 marker system, all donors expressed the CD45.1 marker, whereas all recipients expressed the wild-type CD45.2. Frequency of CD45.1+ splenic IgD-/CD21- B cells, as a fraction of total splenic B cells (B220+/CD19+) is shown, delineated by sex and ST6Gal-1 status of the host. Variation in the size of this compartment was associated with the genotype and sex of the host, with a statistically significant decrease upon loss of host ST6Gal-1 in male, but not female mice. Indicated sample size for each group is given. ** *P* < 0.01.


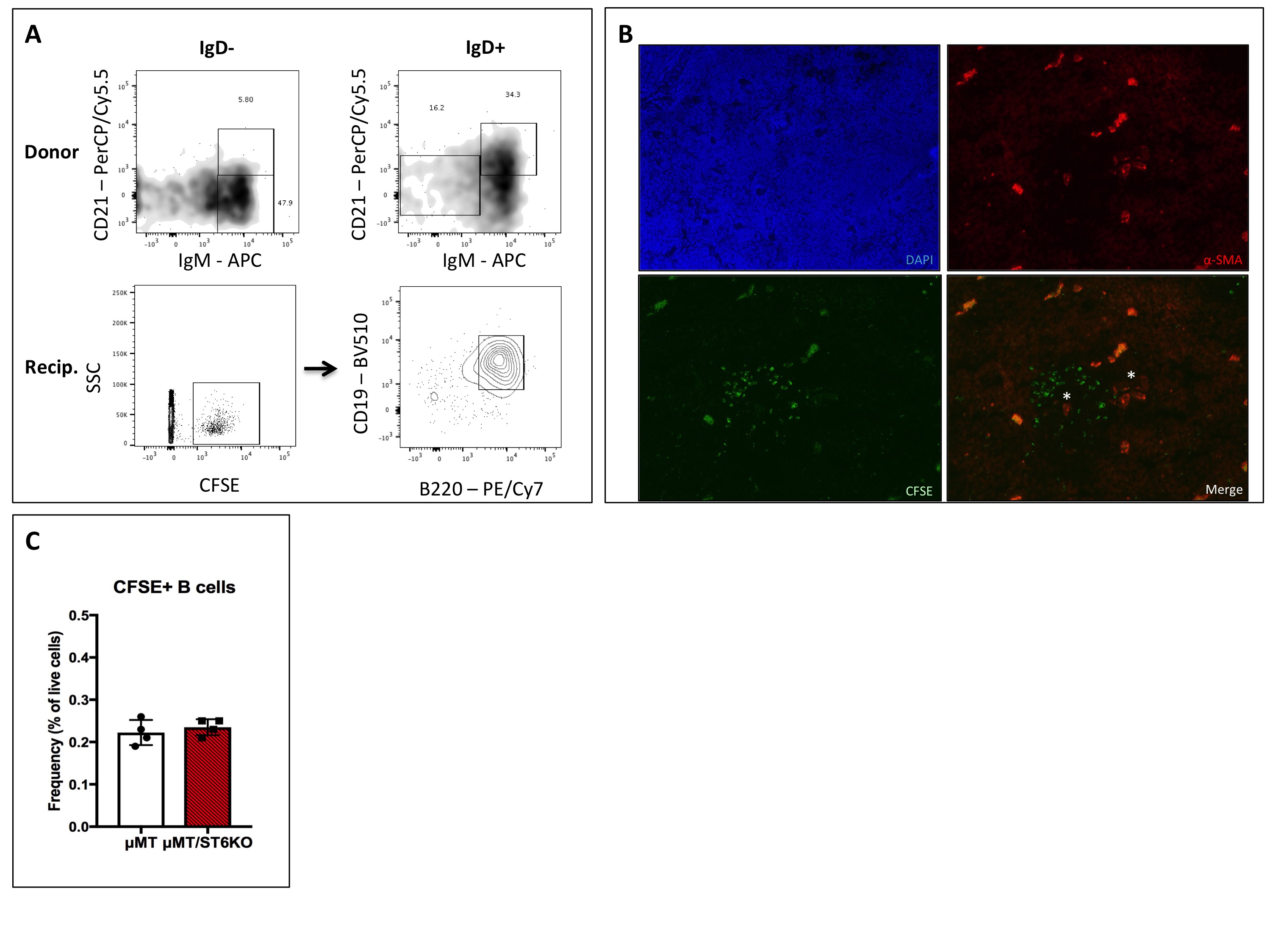


**Supplementary Figure 5. Transitional B cell Migration.** Splenocytes were collected from 6 day old wild-type mice, CFSE-labeled, and intravenously injected into μMT and μMT/ST6KO mice. 24 hours later, recipients were euthanized and spleens analyzed. (A) B220+/CD19+ B cells from day 6 donor spleens are shown. Donor splenocytes consisted of B cells largely limited to IgM-high phenotypes, consistent with transitional B cells, and lacked significant amounts of marginal zone or follicular B cells (top panel). CFSE+ cells were identified in spleens of recipient mice 24 hrs post-injection and found to be largely B220+/CD19+ B cells (lower panel). (B) Migrating CFSE+ cells were found close to arterial structures in the spleen, here identified by presence of smooth muscle actin (red; artery shown with asterisk). (C) B220+/CD19+/CFSE+ B cells were equal between uMT and uMT/ST6 DKO mice. Our results show quantitatively that migration of transitional B cells into the spleen is not impaired at the 24 hour time point, depending on the ST6Gal-1 status of the host.


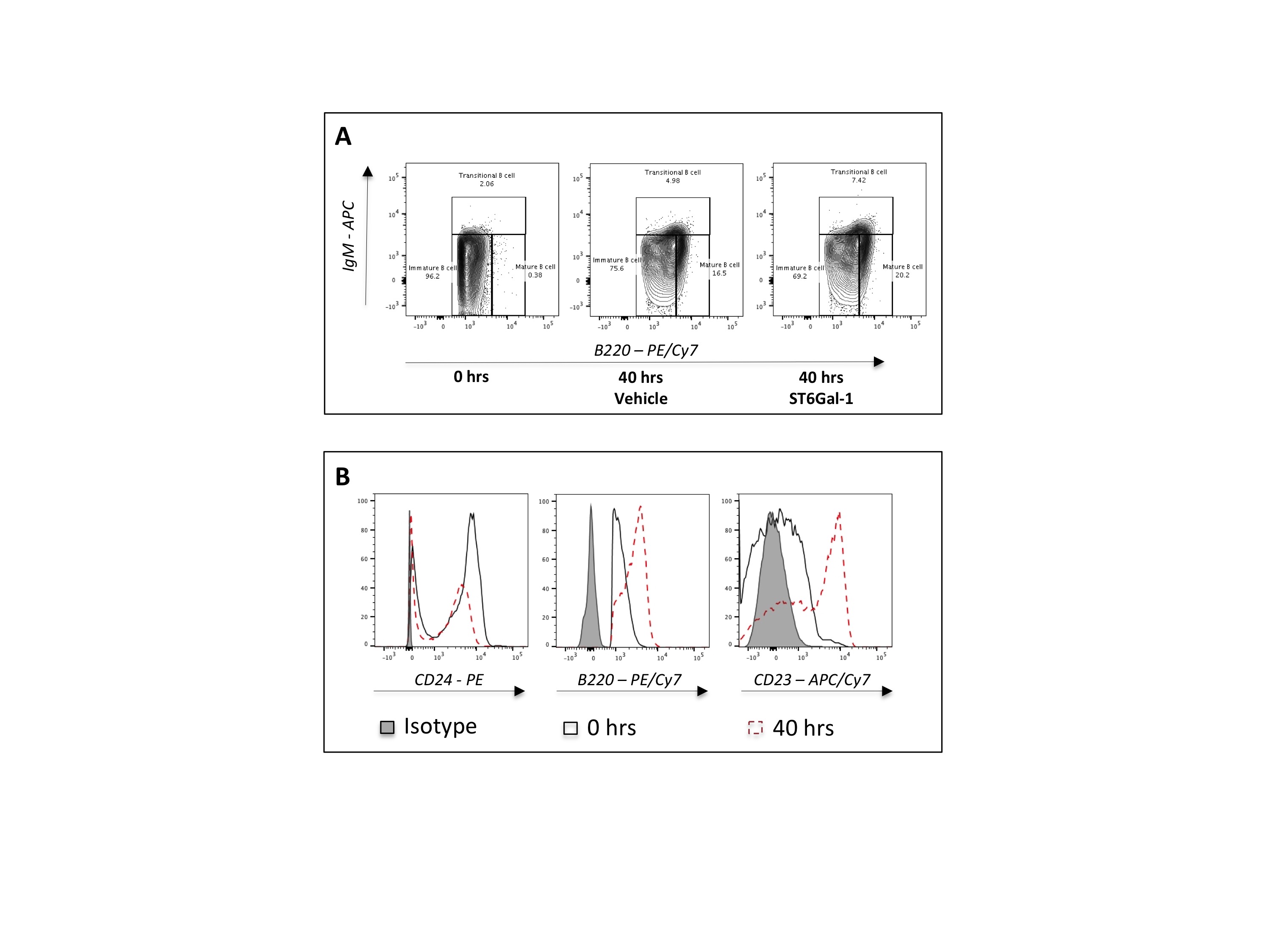


**Supplementary Figure 6. Ex vivo culture of immature B cells.** (A) Phenotypically immature B cells were isolated by magnetic separation and cultured for 40 hours. Representative flow cytometry analysis of B220 and IgM expression is shown, in the presence or absence of recombinant ST6Gal-1. Although populations from *in vitro* culture are not directly comparable with in vivo B cell populations, they are gated identically, and are named immature, transitional, or mature. (B) Representative histograms of CD24, B220, and CD23 expression in total B cells at 0 hrs and 40 hrs (vehicle treated) demonstrates expected downregulation of CD24 and upregulation of B220 and CD23.


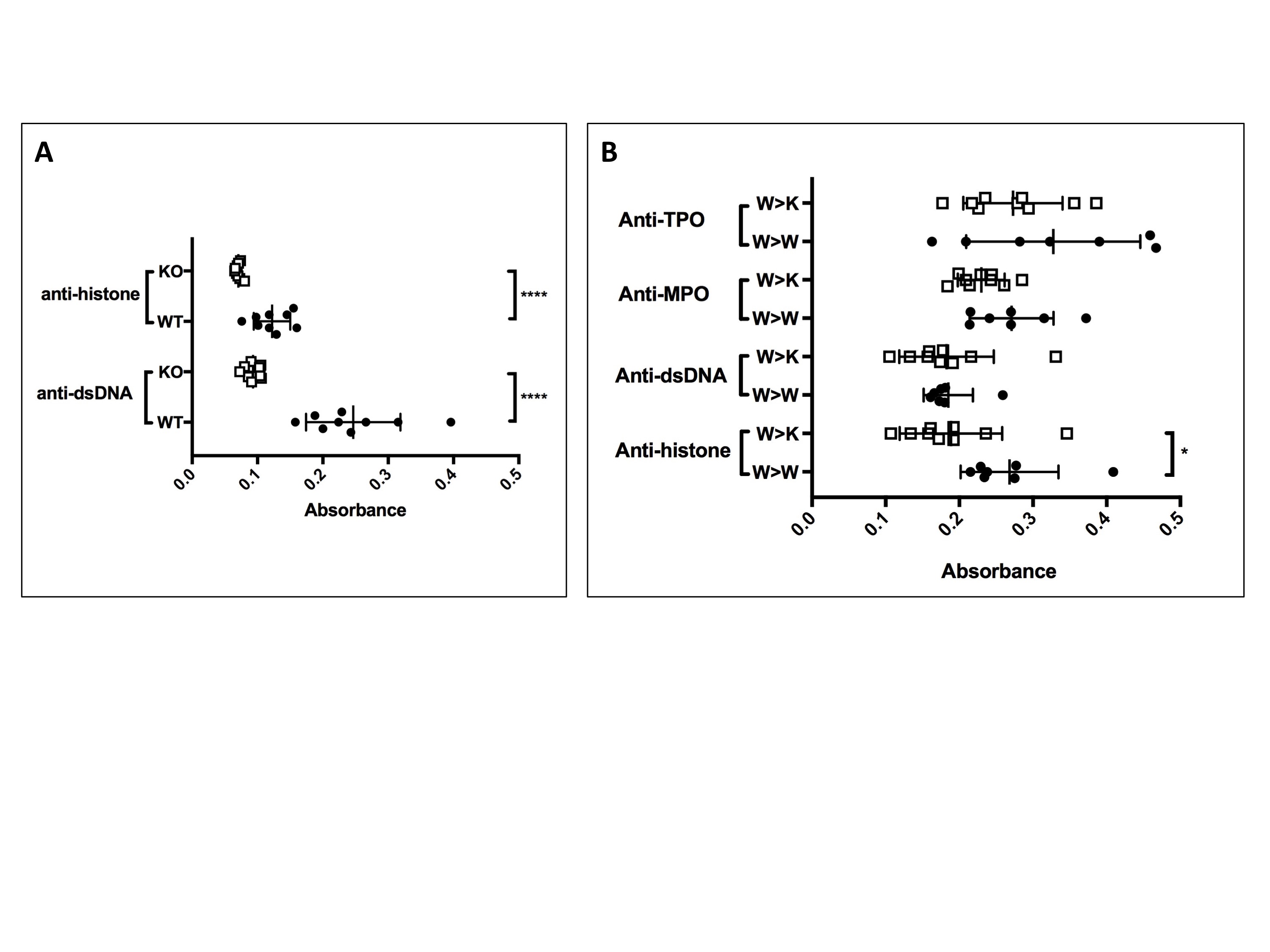


**Supplementary Figure 7. Autoantibody titers in St6gal1-KO mice and chimeras.** Serum from indicated mice was used to detect relative amounts of IgG towards dsDNA, histone, MPO, and TPO by ELISA. (A) Autoantibody titers in wild-type and St6gal1-KO mice are shown. Significant reductions in circulating autoantibodies were observed upon global loss of ST6Gal-1 (n=9). (B) Autoantibody titers in bone marrow chimeras in which wild-type bone marrow repopulated wild-type or St6gal1-KO hosts for 6 weeks (n=8). Anti-histone antibodies were found to be reduced in chimeras in ST6Gal-1 deficient hosts. All other autoantibodies tested did not reach statistical significance. These results suggest that the striking difference in autoantibody titers between wild-type and *St6gal1*-KO mice could not be directly attributed to systemic expression of ST6Gal-1. * *P* < 0.05, **** *P* < 0.0001.


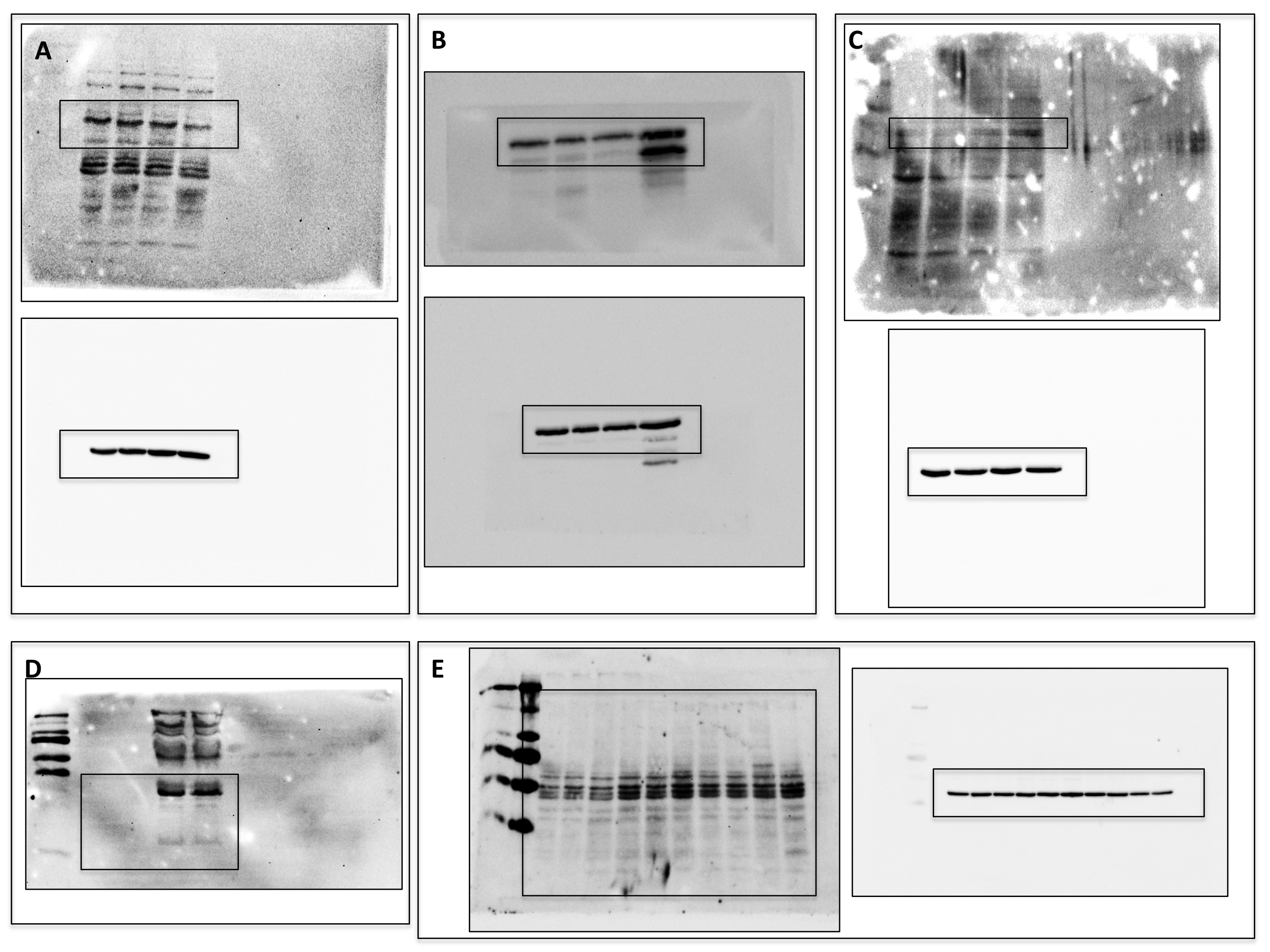


**Supplemental Figure 8. Full-sized immunoblots.** Various immunoblots from Figure 7 are shown, with cropped portion indicated. (A) anti-p100 (top) and anti-B-actin (bottom) from BAFF-stimulated, extrinsically sialylated immature B cells. (B) anti-p-p38 (top) and anti-B-actin (bottom) from BAFF-stimulated, extrinsically sialylated immature B cells. (C) anti-p-AKT (top) and anti-B-actin (bottom) from BAFF-stimualted, extrinsically sialylated immature B cells. (D) anti-BAFFR blot from immunoprecipitation of membrane SNA-reactive proteins of extrinsically sialylated immature B cells. (E) anti-pTyr (4G10) blot (left) and anti-B-actin (right) of extrinsically sialylated immature and transitional B cells stimulated with anti-IgM antibody.


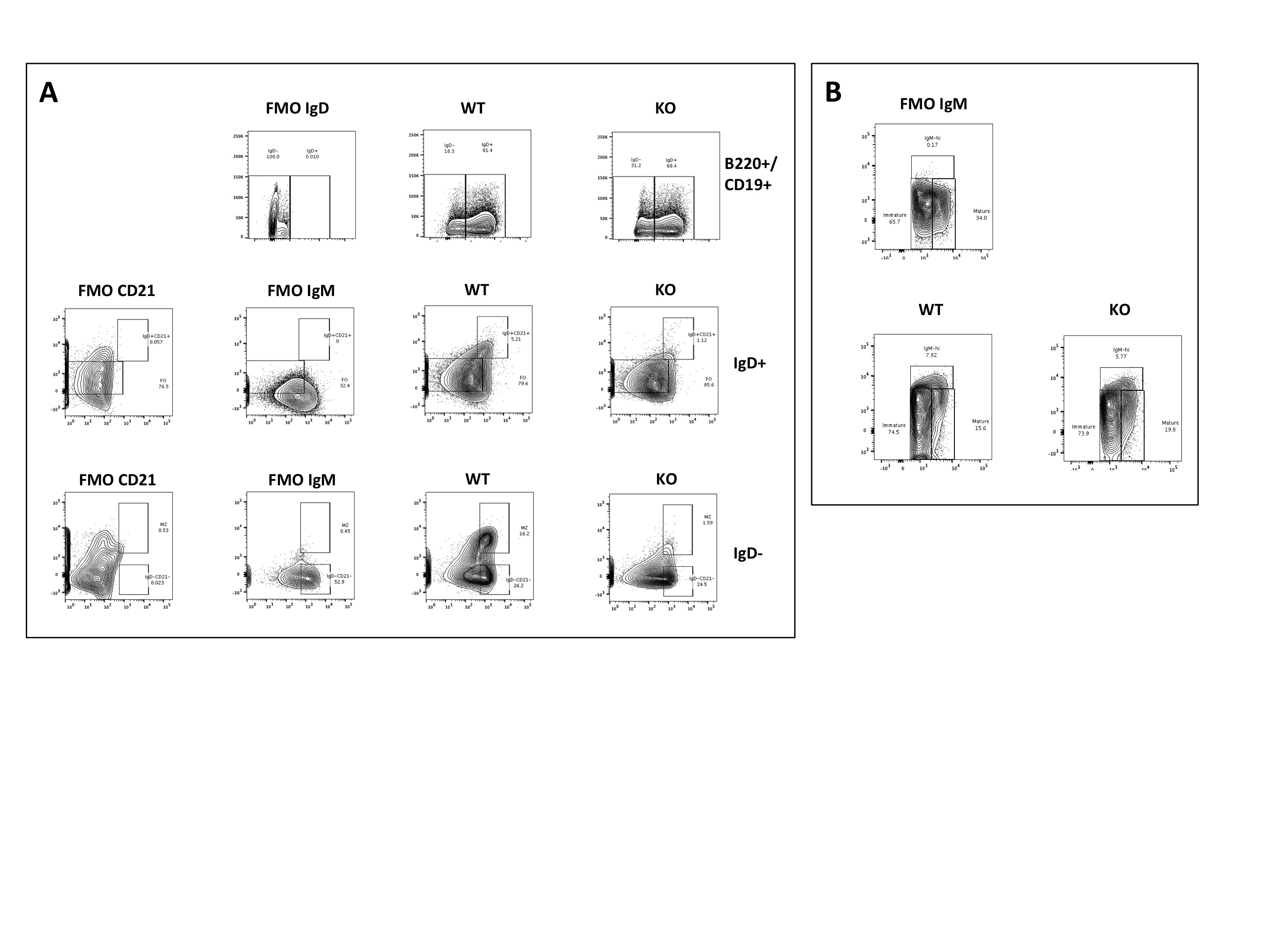


**Supplemental Figure 9. Flow cytometry FMO controls.** (A) Splenic B cells are shown with relevant fluorescence minus one (FMO) controls for IgD, CD21, and IgM. For the latter two, both IgD- and IgD+ B cells are shown with relevant gating used to define cell populations. (B) Bone marrow B cells are shown with fluorescence minus one (FMO) control for IgM.
